# Transcriptomic Profile Reveals Gender-Specific Molecular Mechanisms Driving Multiple Sclerosis Progression

**DOI:** 10.1371/journal.pone.0090482

**Published:** 2014-02-28

**Authors:** Haritz Irizar, Maider Muñoz-Culla, Lucia Sepúlveda, Matías Sáenz-Cuesta, Álvaro Prada, Tamara Castillo-Triviño, Gorka Zamora-López, Adolfo López de Munain, Javier Olascoaga, David Otaegui

**Affiliations:** 1 Multiple Sclerosis Unit, Neuroscience Area, Biodonostia Health Research Institute, Donostia-San Sebastian, Spain; 2 Spanish Multiple Sclerosis Net (REEM), Barcelona, Spain; 3 Hospital Universitario Donostia, Immunology Department, Donostia-San Sebastian, Spain; 4 Bernstein Center for Computational Neuroscience, Humboldt-Universität zu Berlin, Berlin, Germany; 5 Center for Brain and Cognition, Universistat Pompeu Fabra, Barcelona, Spain; 6 Hospital Universitario Donostia, Neurology Department, Donostia-San Sebastian, Spain; 7 Centro de Investigaciones Biomédicas en Red sobre Enfermedades Neurodegenerativas (CIBERNED), Instituto Carlos III, Ministerio de Ciencia e Innovación, Madrid, Spain; University of Jaén, Spain

## Abstract

**Background:**

Although the most common clinical presentation of multiple sclerosis (MS) is the so called Relapsing-Remitting MS (RRMS), the molecular mechanisms responsible for its progression are currently unknown. To tackle this problem, a whole-genome gene expression analysis has been performed on RRMS patients.

**Results:**

The comparative analysis of the Affymetrix Human Gene 1.0 ST microarray data from peripheral blood leucocytes obtained from 25 patients in remission and relapse and 25 healthy subjects has revealed 174 genes altered in both remission and relapse, a high proportion of them showing what we have called “mirror pattern”: they are upregulated in remission and downregulated in relapse or vice versa. The coexpression analysis of these genes has shown that they are organized in three female-specific and one male-specific modules.

**Conclusions:**

The interpretation of the modules of the coexpression network suggests that Epstein-Barr virus (EBV) reactivation of B cells happens in MS relapses; however, qPCR expression data of the viral genes supports that hypothesis only in female patients, reinforcing the notion that different molecular processes drive disease progression in females and males. Besides, we propose that the “primed” state showed by neutrophils in women is an endogenous control mechanism triggered to keep EBV reactivation under control through vitamin B12 physiology. Finally, our results also point towards an important sex-specific role of non-coding RNA in MS.

## Background

Multiple sclerosis (MS) is a complex, chronic and demyelinating disease that affects millions of people worldwide, being the most common chronic disease of the central nervous system (CNS) that begins in early to middle adult life. It is widely accepted that autoimmunity is an important part of the pathological events leading to MS and the oligodendrocytes in the CNS seem to be the main target of the autoimmune response, causing the characteristic lesions in the white matter. Although its etiology is unknown, several genetic and environmental factors have been shown to be associated to the risk of developing the disease. Variants in more than 50 genes [1] and various environmental factors such as smoking, Epstein-Barr virus (EBV) infection and low sunlight exposure have been shown to contribute to the risk [2]. For most of these factors the association is modest and, thus, the general view is that the interaction of many of these risk elements in an individual is what leads to the development of MS.

The most common clinical presentation of the disease (in 85–90% of the cases) is the so called Relapsing-Remitting MS (RRMS). In this form of the disease patients suffer from episodes of acute onset of neurological symptoms that typically last from 48 hours to several weeks called relapses followed by longer periods of clinical stability. This transient nature of neurological symptoms is explained by subsiding inflammation, remyelination of axons and the plasticity of the nervous system. Inflammation is associated with MS lesions and it has been postulated to be the cause of, or alternatively a reaction to, axon demyelination and damage [3]. After a relapse, a reduction of neurological disability happens but, sometimes, the recovery is not complete and thus, with every new relapse, neurological disability is accumulated. The recovery of neurological ability after a relapse happens mostly due to remyelination of the lesions caused by the immunological attack.

Most of the treatments currently used are immunological agents aimed to reduce the inflammatory response and the clinical and neurological activity in order to delay the progression of the disease. However, the use of these immunomodulatory and immunosupressor treatments has encountered several problems such as low efficacy and high-proportion of non-responders (interferon beta-1a, interferon beta-1b and glatiramer acetate) or severe secondary effects (natalizumab and mitoxantrone). Basically, the problem resides on the fact that these treatments modulate general non-MS-specific immunological processes, such as immune-cell infiltration to the CNS (natalizumab) or inflammation (interferon beta and glatiramer acetate). The lack of knowledge on the MS-specific molecular and cellular events behind the relapses and driving disease progression has hampered the design of efficient treatment strategies.

In general terms, the relapses are thought to be a consequence of T-cell driven inflammatory processes, with massive infiltration of leukocytes into the CNS through blood-brain barrier (BBB) disruption. Some evidence also points to a role of B cells in the progression of the disease [4]. However, the molecular mechanisms underlying these pathogenic cellular inflammatory responses have not been properly described. Although some factors have been proposed to exacerbate the pathogenic inflammatory response, relapse episodes are still unpredictable. A deeper understanding of the MS-specific processes responsible for the relapsing-remitting behavior of the immune system and a description of the molecular events that trigger the inflammatory response in relapses would provide a molecular framework for the design of more effective and safer treatment strategies.

Currently, there is no molecular model for MS; there is even no consensus on the molecular events playing a central role in the pathogenesis of the disease. To tackle this problem, we perform here a hypothesis-free approach to investigate the possible molecular events responsible for the progression of RRMS. A whole genome gene expression analysis of peripheral blood leukocytes at the RNA level of patients in remission and relapse has been performed in order to draw as complete as possible a picture of the molecular mechanisms responsible for the progression of RRMS.

## Results

### Differential Expression Analysis

For the differential expression analysis the samples were grouped based on their treatment state:

- All samples (68, “All” group)
- Samples from patients with the same disease-modifying treatment in relapse and remission and the matched control samples (39; “Same treat” group)
- Samples from patients with no treatment both in relapse and remission and the matched control samples (12, “No treat” group).

It must be taken into account that each sample set is a subgroup of the previous set, not an independent group. Two comparisons were made for each sample set: relapse vs. remission and remission vs. controls. Besides, three analyses were performed in parallel: all the samples, female samples and male samples. Finally, two algorithms were used for the comparisons: t-test and Rank Products. When checking for differences between relapse and remission, paired comparisons were done. This resulted in 36 comparisons that produced 36 differentially expressed gene-lists.

**Figure 1:**
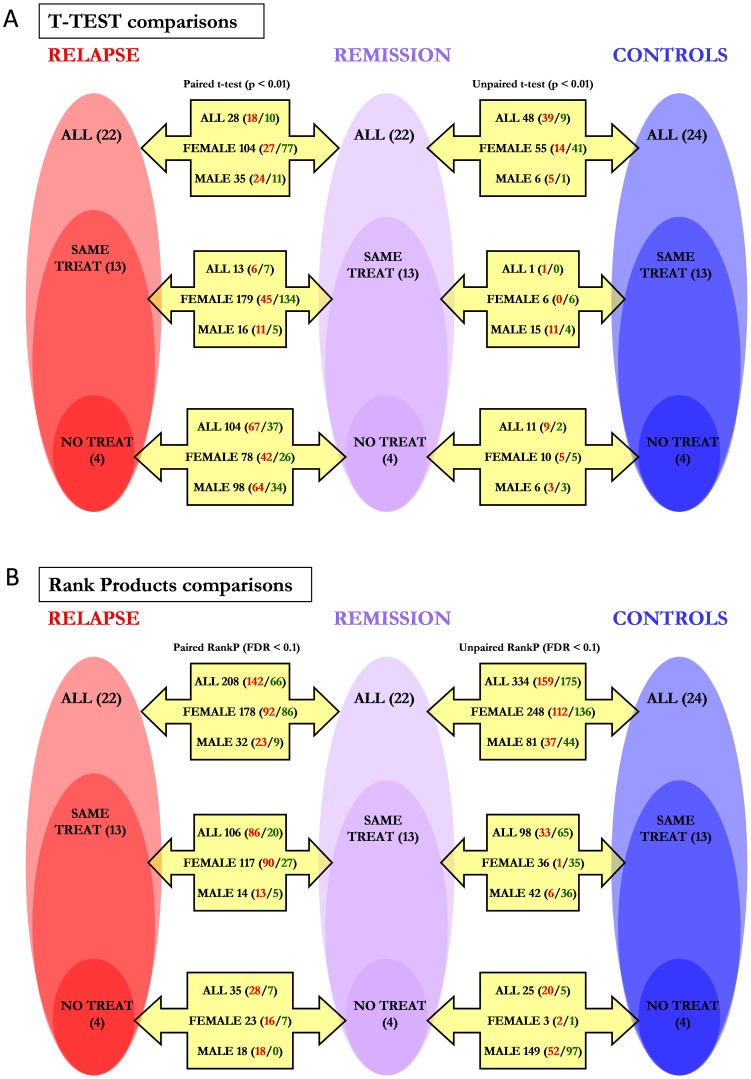
Differentially expressed gene-lists obtained in the 36 comparisons. The samples have been grouped based on the treatment state (all samples (n = 68), samples with the same treatment in remission and relapse (n = 39) and samples with no treatment at all (n = 12)) and gender (all samples, female samples and male samples) and two gene expression comparisons (relapse vs. remission and remission vs. controls) have been performed within each group using two algorithms: t-test (uncorrected p<0.01) and Rank Products (FDR <0.1). Paired comparisons have been done for relapse/remission comparisons as the samples were obtained from the very same patients. The length of the differentially expressed gene-list for each comparison is shown in bold. The distribution of over- and underexpressed genes is also showed (**all genes/overexpressed genes/underexpressed genes**). **A**: lists obtained with the t-test algorithm; **B**: lists obtained with the Rank Products algorithm.

The number of genes of the lists and the distribution of over- and underexpressed genes in each case can be seen in **figure 1**. In total, 1167 unique probesets have been shown to be differentially expressed and the average number of genes per list is 71. The average gene number and the average over−/underexpressed gene ratio for each of the comparison types are shown in **table 1**. The Rank Products algorithm has been shown to create (with a p value <0.05), on average, longer lists than the t-test analysis. Besides, the average number of differentially expressed genes has been significantly lower in the comparisons with the “Same treat” sample set than in the comparisons with the “All” sample set. Finally, the over−/underexpressed gene ratio (up/down ratio) is significantly lower in the comparisons made with female samples than in the comparisons made with all the samples. No other interaction between comparison type and these two gene-list characteristics has been detected.

**Table 1:** Average gene number and upregulated/downregulated gene ratio per list by comparison type (based on algorithm, sex, treatment and disease state).

	Genes per list	Up/down gene ratio
Comparison type	(mean ± std(p value))	(mean ± std (p value))
T-test*	**45.17**±49.08	**1.87**±1.52
Rank Products	**97.06**±93.54 (**0.043**)	**1.88**±1.41 (0.856)
All*	**84.25**±98.41	**2.65**±1.59
Females	**86.42**±80.45 (0.911)	**1.20**±1.01 (**0.004**)
Males	**42.67**±44.32 (0.166)	**1.97**±1.35 (0.175)
All*	**113.08**±104.59	**1.86**±1.50
Same treat	**53.58**±57.25 (**0.039**)	**1.71**±1.52 (0.892)
No treat	**46.67**±48.19 (0.096)	**2.41**±1.38 (0.324)
Relapse vs remission*	**77**±63.36	**2.08**±1.11
Remission vs controls	**65.22**±92.17 (0.526)	**1.69**±1.73 (0.432)

*The group used as reference for the T-test comparisons in each case.

As the total number of differentially expressed probesets was high (1167) and the comparisons were made in a non-stringent manner, we decided to reduce the total gene list by matching the lists obtained in each of the relapse vs. remission and remission vs. controls comparison-pairs in order to detect the most relevant genes from a pathophysiological point of view, assuming that if a gene is altered both in relapse and remission the probability of it having a relevant role in the disease increases dramatically. There were 18 comparison pairs and, thus, 18 gene-lists were obtained with genes altered both in relapse and remission. Only two genes were obtained from the match-up of the lists produced by the t-tests; in contrast, 172 genes were found to be altered both in relapse and remission from the lists produced by the Rank Products, for a total of 174 genes. Seven of the nine gene list match ups performed with t-test based lists resulted in no common genes and, thus, just 11 lists were obtained with 1 or more common genes (**Figure S1**).

Then, these genes were classified into three groups:

- *Female genes*: genes that appeared in a female comparison but not in a male comparison.
- *Male genes*: genes that appeared in a male comparison but not in a female comparison.
- *Common genes*: in the rest of cases.

The list of common and male genes with the fold-changes for each comparison can be seen in **Table S1** and the list of female genes in **Table S2**. The properties of the lists of each specificity (female/male/common genes) are shown in **table 2**. It stands out the high proportion of genes altered in the opposite direction (upregulated in relapse/downregulated in remission or downregulated in relapse/upregulated in remission) in all the groups, a proportion that is higher than expected by chance in a statistically significant manner (p<0.001 in all groups). Thus, it seems that the alteration of gene expression sustained in remission is reversed in relapse. We have called this the “**mirror pattern**”.

**Table 2:** Number of genes differentially expressed in both conditions by specificity (female, male, common), proportion of genes altered in the opposite direction in relapse and remission and significance of this proportion (p value of chi-square test).

	Female genes	Male genes	Common genes	All genes
**Gene number**	104	12	58	174
**Genes opp. direction (%)**	97 (93.3%)	12 (100%)	43 (74.1%)	152 (87.4%)
**χ^2^ p value**	<0.0001	0.0005	0.0002	<0.0001

### Coexpression Analysis

**Figure 2:**
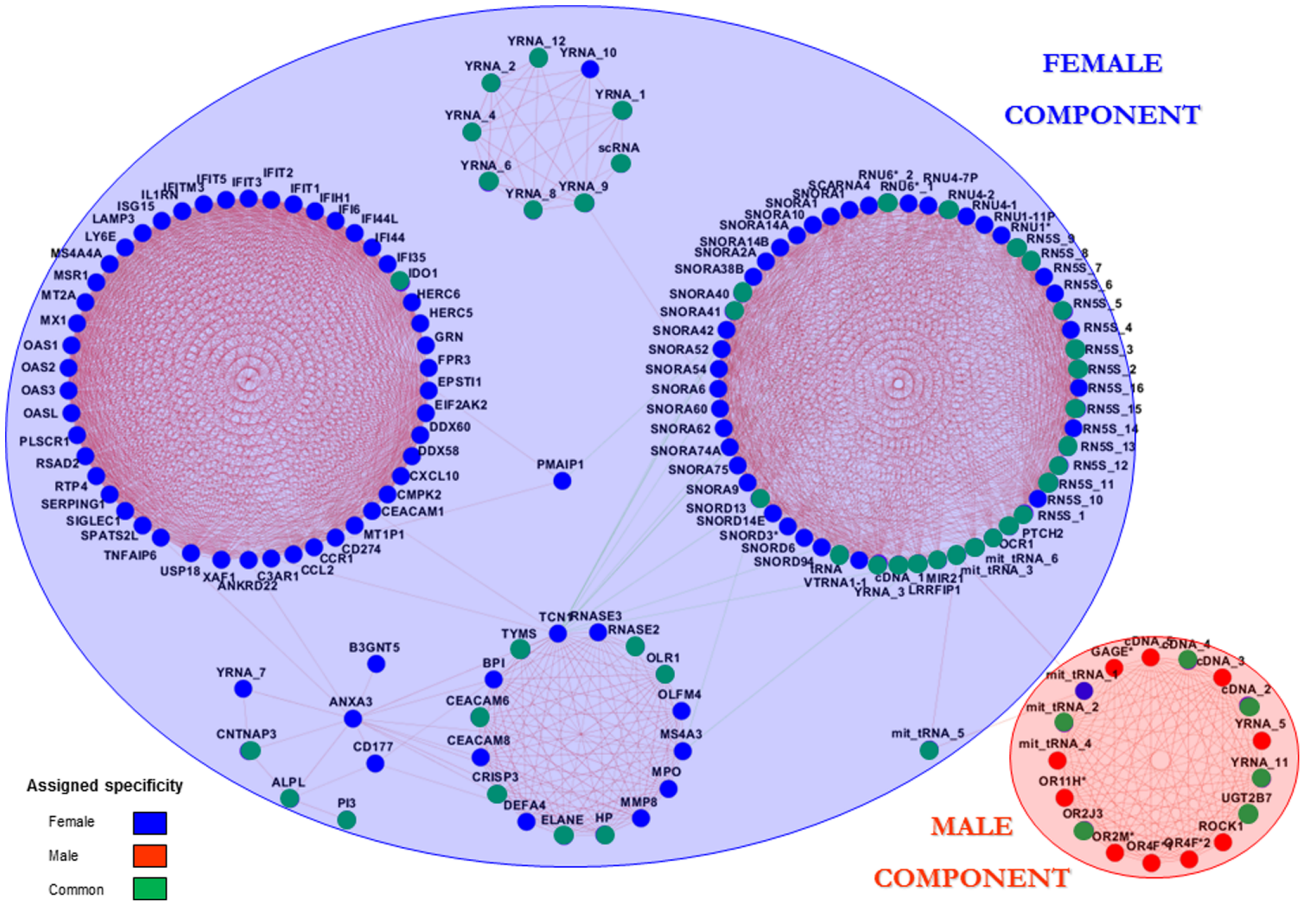
Results of the coexpression analysis performed on the 174 differentially expressed genes. The main component of the network is shown with a visualization of the specificities (female/male/common) previously assigned to the genes. **Blue nodes**: female genes; **red nodes**: male genes; **green genes**: common genes. **Red edges**: positive correlations; **green edges**: negative correlations.

In the following we studied the relation between the expression patterns of the genes, in order to find groups of genes with similar behavior and the relations between them. For that, a coexpression network was built. We first created a coexpression matrix based on the expression values of the 174 genes across the 68 samples by computing pairwise Pearson’s R between the genes and it was subsequently cut at R>>|0.6| (p<10^−7^). The resulting network was visualized in Cytoscape (**figure 2**). 157 out of the 174 studied genes are part of a fully connected component, where a clear separation of gene-clusters can be seen. The distribution of the genes suits the female/male gene classification previously done, with the exception of a *female gene* that shows higher connectivity with *male genes*. From the genes previously classified as common, 44 appear in the part of the network we have designated as *female component* and six appear integrated in the *male component*. The 140 genes that comprise the female component may be involved in or be relevant to female specific events happening in leukocytes of patients. Likewise, the 17 genes of the male component may have important roles in the molecular pathogenic processes acting in male patients.

**Figure 3:**
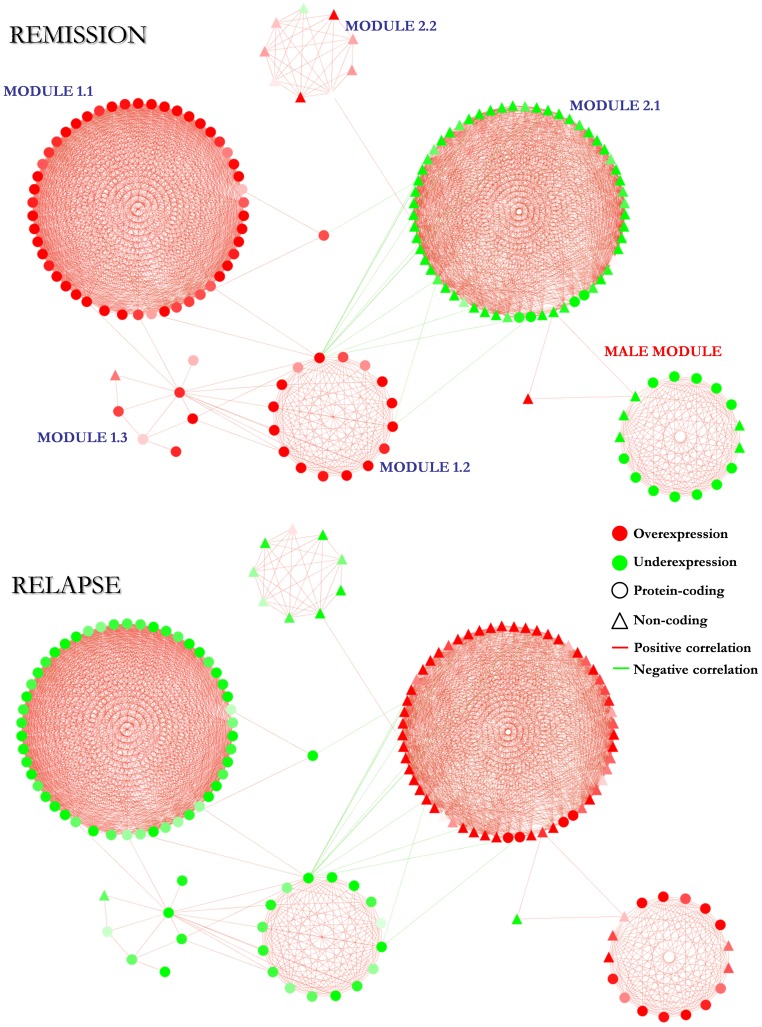
Main component of the coexpression network with the fold-changes obtained in remission and relapse. The color of the nodes codes for the differential expression of the genes (overexpression/underexpression), the node shapes for the type of gene (protein-coding/non-coding) and the color of the edges for the sign of the correlation (positive correlation/negative correlation).

The fold-changes that these genes have in different conditions (relapse vs remission; and remission vs controls) have been visualized in the network (**figure 3**). Interestingly, the mirror pattern previously described is maintained in virtually all the genes (156/157 (99.37%)) keeping a homogeneous behavior inside each of the four main modules (the 1.1, 1.2 and 2.1 female modules; and the male module) as expected. In these modules, the 99.29% of the genes shows the mirror pattern and within each of them all the genes have the same behavior (up in relapse/down in remission; or down in relapse/up in remission) with the exception of 1 gene in the female module 2.1. A list of the genes of each of the four main modules with the corresponding fold-changes and assigned specificities can be found at **Table S3**.

**Figure 4:**
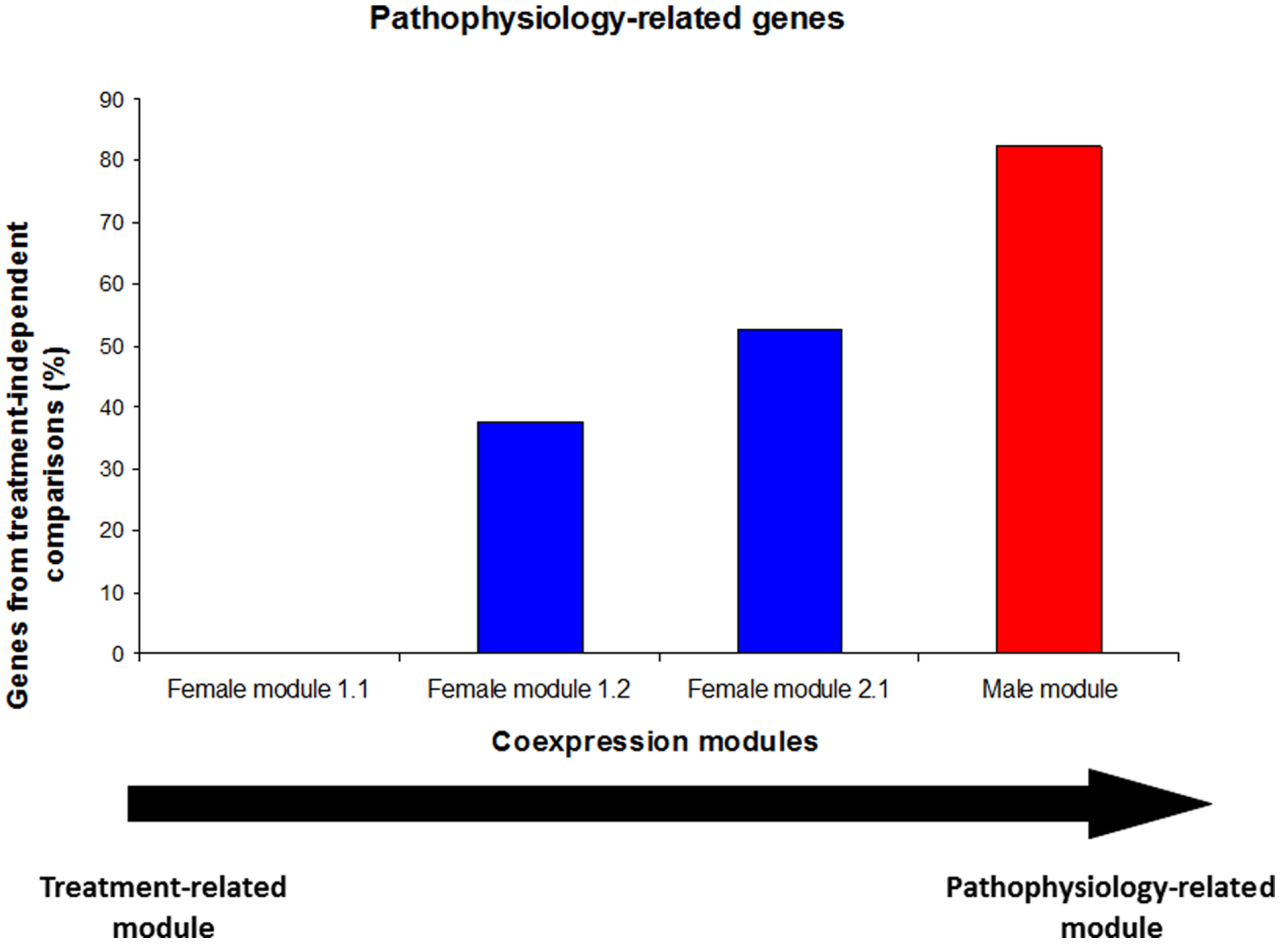
Percentage of genes obtained from “treatment-independent” comparisons for the main coexpression modules. The percentage of the genes that appeared in the comparisons made with the samples from the patients with the same treatment in remission and relapse and from patients with no treatment at all is visualized and used as a measure for how truly a module reflects the underlying pathophysiology.

Regarding treatment, three comparison types have been performed as described before: “all” comparisons, “same treatment” comparisons and “no treatment” comparisons. Alterations in the gene expression obtained from the “all” comparisons, could be explained, at least partially, by the different treatment that the patients were receiving during the relapse and remission phases. This could reflect the implication of these genes in the effect of treatment more than in the pathophysiological processes happening in the disease. The genes obtained from the other two comparison types (“same treatment” and “no treatment”), where the effect of a different treatment is eliminated, can be assumed to be more strongly related to endogenous mechanisms of the disease. We call these last two comparison types “treatment-independent” comparisons. The percentage of genes obtained from “treatment-independent” comparisons has been calculated for the 4 main modules of the coexpression network (**figure 4**). A gradient can be observed in the capacity of reflecting the pathophysiology of the disease from the female module 1.1 (it reflects just the effect of treatment) to the male module (nearly completely linked to disease pathophysiology). The other two female modules show an intermediate capacity of reflecting the pathophysiological events occurring in peripheral blood leucocytes of patients with multiple sclerosis. It has to be noted that the fact that the male module reflects the underlying pathophysiology better than the female modules is probably a consequence of all but one untreated patients (untreated both in remission and relapse) being males.

In order to describe the molecular/cellular mechanisms reflected by the coexpression modules, biological information on the genes of the four main modules has been collected and visualized (**Figure S2**). On one side, several searches have been performed on databases (NCBI’s Entrez Gene, GeneCards and KEGG pathways) and literature looking for information about molecular/cellular function and tissue distribution of gene expression. Among all that information, the term describing the aspect that, in the context of the present work, we believe is most relevant has been chosen for each gene. On the other side, making use of the DAVID GO online tool, a biological term enrichment analysis has been performed. Finally, all this information, along with the gene expression fold-changes for each condition and the percentage of the genes of each specificity (female/male/common), has been visualized.

As it can be seen in **Figure S2**, most of the genes (61%) of the female module 1.1 are virus/interferon response genes and show an “up in remission/down in relapse” pattern. This module, as has been shown in **figure 4**, probably reflects more the effect of treatments than the pathophysiological processes underlying the disease. In the female module 1.2, the vast majority of the genes (88%) are genes expressed in neutrophils and eosinophils and show the same expression alteration pattern (up in remission and down in relapse) as the genes in the module 1.1. In this case, the 37.5% of the genes were obtained from treatment-independent comparisons (**figure 4**) so, the mechanism represented by this module (eosinophil/neutrophil activation) may be linked to both the effect of treatments and the pathophysiological events happening in the disease. In the female module 2.1, the 94.7% of the genes are non-coding genes involved in post-transcriptional control of gene expression (49.1%) and ribosomal RNA modification (42.1%) and show the opposite pattern (down in remission/up in relapse) to that of the genes of the previous modules. In this case, half of the genes were obtained from treatment-independent comparisons, so as with the module 1.2, the mechanisms represented in this module (RNA modification and post-transcriptional control of gene expression) may be linked both to treatment effects and pathogenic processes. Finally, the only coexpression module classified as male-specific shows a composition of genes with apparently unrelated functions, with high proportions of protein-coding genes with unknown function (29.4%), genes belonging to the olfactory receptor family (29.4%) and regulatory non-coding genes (29.4%) as main features. As in the female module 2.1, the alteration pattern of the genes of this module is “down in remission/up in relapse”. This module is the one most faithfully reflecting the underlying pathogenic processes going on in the disease (**figure 4**) and, thus, although it is the most functionally heterogeneous module and the one where deducing the reflected mechanism is most difficult, the genes involved in the module may have relevant roles in the pathophysiology of the disease in male patients.

With the aim of elucidating the effect of treatment and sex in each of the modules, data subsets have been created based on these two variables (**treatment state**: different treatment samples, same treatment samples and no treatment samples; **sex**: females/males/all) and two parameters have been calculated for each gene: **1– mean p^2^** and the **mean accumulated deviation.** These have been plotted to detect in which of the data subsets each module presents the most marked and stable alteration of gene expression (**Figure S3**). Five data-points stand out the rest in terms of deviation and significance. The deregulation of module 1.1, as expected, has its source in the female samples with different treatment in remission and relapse and, thus, it is clear that it reflects the effect of treatments on the system. The alteration of the genes of the 1.2 and 2.1 modules, instead, mostly comes from the female samples with the same treatment in remission and relapse, indicating that they are reflecting female-specific mechanisms related to the pathophysiology of the disease. Finally, the genes of the male module seem to be altered more prominently in the male samples with no treatments than in the rest of sample subsets and, thus, the molecular processes represented by this module are male-specific and pathophysiology-related. Although no clear conclusion can be made for the 1.3 and 2.2 modules, these results confirm the previous assigned sex-specificities of the modules and their relations with treatments/pathophysiology.

In order to identify the most relevant genes from the point of view of the network structure we computed the betweenness-centrality of each node in the female component (**Figure S4**). The betweenness quantifies how central a node is among the paths of communication in the network. If a node has betweenness equal to one, it means that all communication paths pass through this node. A node with zero betweenness is irrelevant for the transmission of information within the network. Transcobalamin 1 (TCN1) is the gene with the highest betweenness-centrality, as 38.3% of the shortest paths between all node-pairs of the network go through this node. The removal of this gene causes a drop in the connectivity of the network and an increase in the mean shortest distance (from 3.187 to 3.420). These results indicate that the TCN1 gene, involved in the transport of cobalamin (vitamin B12) into cells, may have an important regulatory role in the molecular events reflected by the female component.

### The “Mirror Pattern”

**Figure 5:**
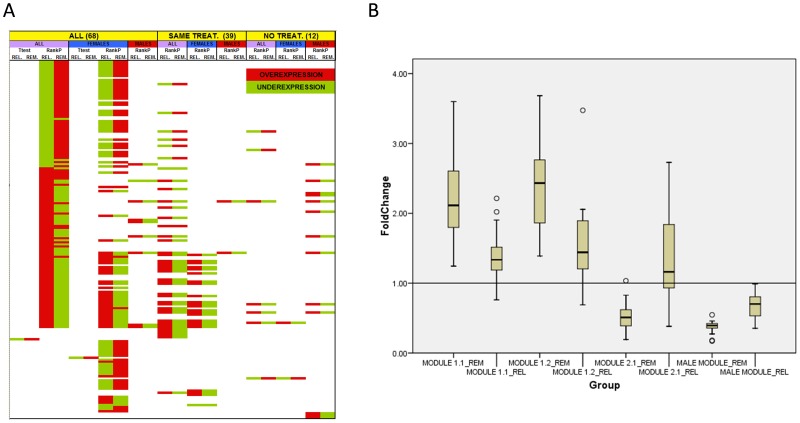
Graphical representation of the “mirror pattern” of the genes altered both in relapse and remission. **A**: direction of the alteration (over/underexpression) of the 174 differentially expressed genes in the different comparisons. **REL**: relapse vs. remission; **REM**: remission vs. controls. **B**: box-plot representation of the fold-change values vs. controls in remission and relapse for each of the four main modules of the coexpression network. **_REM**: remission vs. controls; **_REL**: relapse vs. controls.

As previously shown, a high proportion of the differentially expressed genes are altered in the opposite direction in remission and relapse, a behavior that gives rise to a pattern that we have designated as “mirror pattern”. A visual representation of this behavior and its maintenance across different comparisons can be observed in **figure 5A**. However, obvious questions arise from the mirror pattern: how strong is the effect of relapse at the reversal of the gene expression pattern maintained during remission? Are reference expression values provided by controls approached or surpassed? Does the intensity of this effect change between the modules of the coexpression network? In order to tackle these questions, fold-changes against control values of both remission and relapse have been box-plotted for the four largest modules of the network (**figure 5B**).

As can be observed, in the 1.1 and 1.2 female modules and the male module, the expression values of most of the genes in relapse approach the reference value set by the controls but they do not surpass it in the opposite direction, except in some few cases. Instead, in most of the genes of the female module 2.1, the expression values do surpass the control reference in the opposite direction. According to this, it seems that the expression pattern-reversing effect of the relapse is stronger in the female module 2.1 than in the rest of the modules of the coexpression network.

### Tcn1 Concentration in Serum

**Figure 6:**
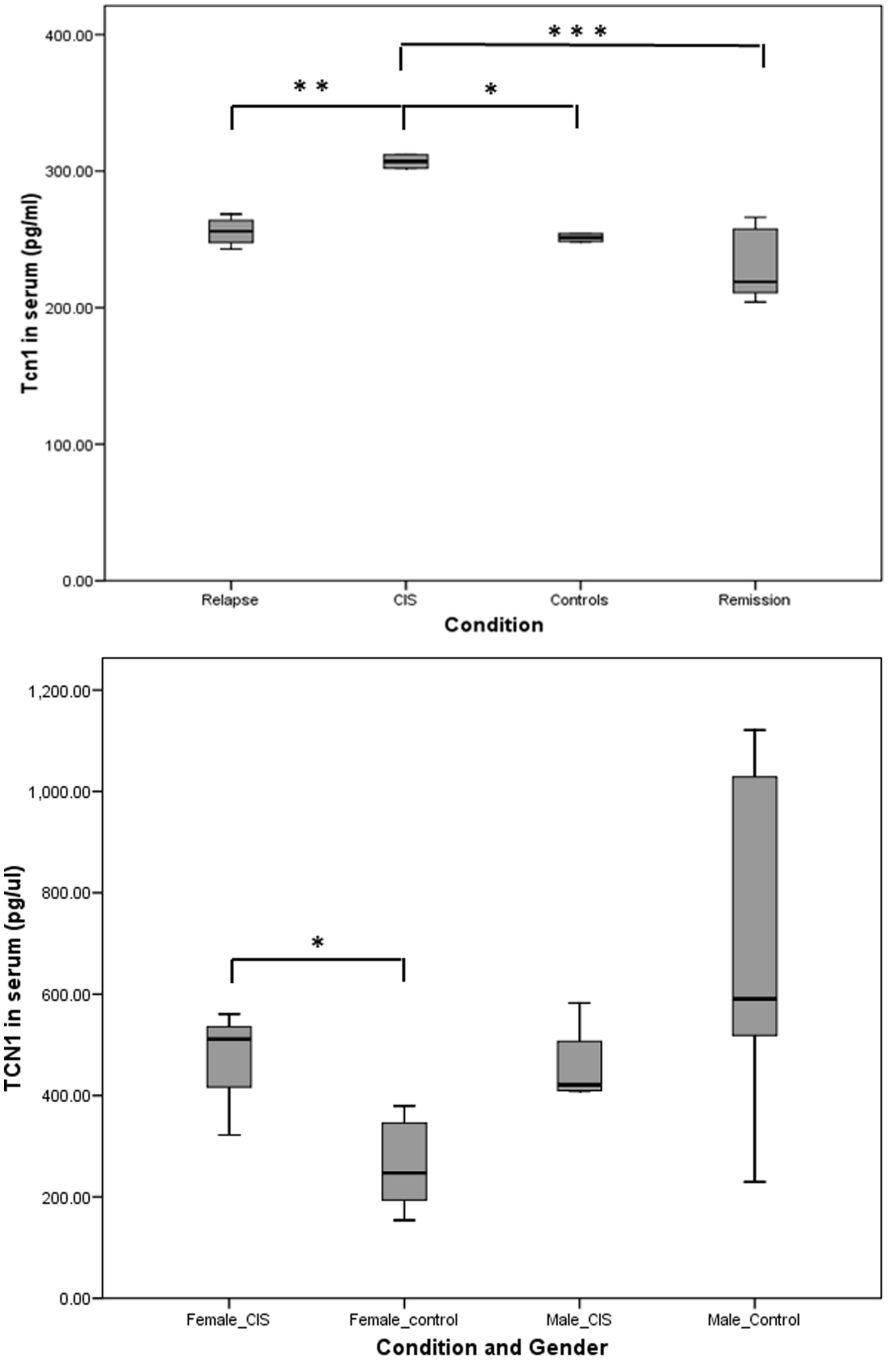
Serum concentration of Tcn1 of patients in remission, relapse and CIS and healthy controls. **A:** box-plot of the protein concentration values in the four groups of the first sample set (49 samples). **B:** box-plot of the protein concentration values of the samples of the second set (19 samples). *p<0.05; **p<0.01; ***p<0.001.

The network topology of the coexpression network has revealed a putative regulatory role for the TCN1 gene (**Figure S4**). Tcn1 is a secreted protein implicated in the transport of vitamin B12 into cells and, in our network, it appears coexpressed with neutrophil-specific genes, suggesting that is produced and secreted by this cell type. Our data shows that TCN1 expression is deregulated in remission and relapse at the RNA level, but as, for this gene, it is the protein product the functional molecule we decided to test its concentration in the sera of MS patients and healthy controls. Tcn1 concentration has been measured in an initial set of 49 serum samples (17 healthy controls, 18 patients in remission, 10 patients in relapse and 4 subjects diagnosed as Clinically Isolated Syndrome (CIS)). No statistically significant differences have been found between controls, patients in remission and patients in relapse (**figure 6A**). However, the four patients with CIS show a statistically higher concentration of serum Tcn1 when compared to the other groups. To confirm these differences, a second set of 19 samples (12 healthy controls and 7 patients with CIS) has been analyzed (**figure 6B**). These results confirm a significantly higher concentration of Tcn1 in female patients, but not in male patients, supporting the idea of transcobalamin 1 having a role in the pathophysiology of MS in females, at least at the initial stages of the disease.

### EBV Gene Expression

**Figure 7:**
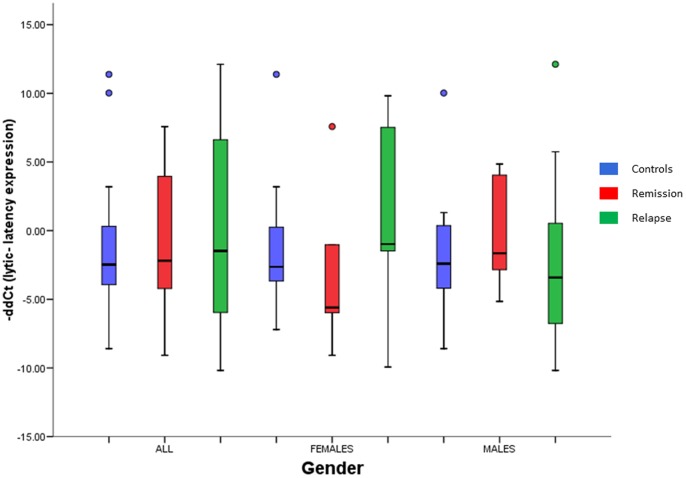
Viral gene expression of the EBV in peripheral blood leukocytes. -ddCts of lytic vs. latency gene expression were obtained from –dCts of BZLF1 expression (lytic expression) and the mean –dCts of the 4 latency genes (EBNA1, EBNA2, LMP1 and LMP2; latency expression). -ddCt >0: higher lytic expression; -ddCt <0: higher latency expression. None of the differences between conditions (controls, patients in remission, patients in relapse) were statistically significant at p<0.05 in none of the groups (all samples, female subjects and male subjects).

As the main hypothesis behind the deregulation of the female module 2.1 and the male module is a reactivation of the Epstein - Barr Virus infection in B cells (see discussion), the expression of 5 viral genes (BZLF1, EBNA1, EBNA2, LMP1 and LMP2) has been measured by qPCR to test it. A -ddCt has been calculated between BZLF1 expression (lytic expression) and the mean expression of the 4 latency genes (latency expression) for female samples and male samples (**figure 7**). The results show the opposite lytic/latency expression pattern in females and males. Viral gene expression shows a more latent profile in female patients in remission than in female controls and shifts to a lytic pattern in relapses, supporting the idea of an EBV reactivation in relapse. In males, on the contrary, the viral gene expression is more lytic in remission and more latent in relapse, contradicting the hypothesis of a reactivation of the virus in relapses of male patients. Nevertheless, it has to be noted that none of the differences is statistically significant at p<0.05.

**Figure 8:**
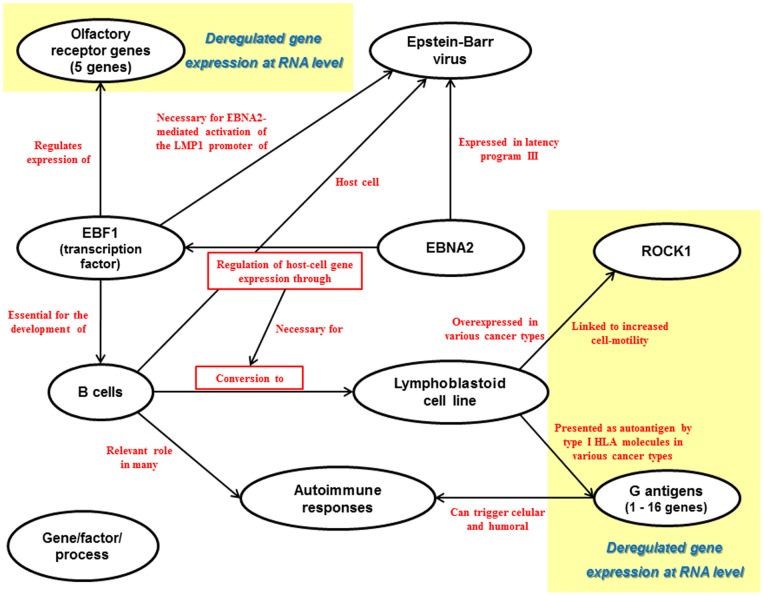
Functional relations between some of the genes of the male module and processes, cells and events known to be involved in multiple sclerosis as described by literature.

Besides, the rationale behind the EBV reactivation hypothesis in males is an overexpression of EBNA2 in relapses that could explain the deregulation of the olfactory receptor genes, ROCK1 and the G antigens (see **figure 8**). EBNA2 expression, however, shows the opposite pattern (FC_remission/controls_: 2.29; FC_relapse/remission_: - 3.30) and, thus, does not support the idea of its overexpression being the cause of the upregulation of the genes of the male module in relapses.

## Discussion

The whole-genome gene expression analysis performed with the objective of the identification of the molecular mechanisms responsible for the progression of RRMS has identified 174 genes whose expression has been found to be altered both in relapse and in remission. The coexpression analysis of these genes has detected several gene-groups organized in coexpression modules that reflect gender-specific molecular processes implicated in the disease. Virtually all these genes present the behavior we have called mirror pattern: if a gene is overexpressed in remission is underexpressed in relapse and vice versa.

### Coexpression Modules

#### Module 1.1: virus and interferon response

As previously mentioned, 61% of the genes of this module are interferon and/or virus response genes and, thus, this module is clearly reflecting the effect of interferon-treatment on leukocyte gene-expression. Besides, all of the genes present the differential-expression pattern we called “mirror pattern”. However, in this case, the mirror pattern is the result of some relapse and remission samples having different treatment states and is not linked to the endogenous molecular dynamics implicated in the progression of the disease. From the 22 MS patients included in the analysis, five were receiving interferon treatment (Rebif or Betaferon) when the remission sample was collected, while either they had no treatment in relapse or were on natalizumab. The fact that in the five sample trios (relapse/remission/control), while the remission sample was under the effect of interferon-treatment, neither the relapse sample nor the control sample were under this effect explains both the differential expression of this genes and the mirror pattern they show.

On the other side, this module has been linked specifically to women. However, of the five patients that we have just mentioned, four are women and, thus, when performing the comparisons with males regardless of treatment-state, just one sample with the interferon in remission/no interferon in relapse pattern was included, making it impossible to observe the effect of interferon treatment on males. Therefore, it is clear that the treatment with interferon alters the expression of these genes in females, but it can’t be concluded that this effect is female-specific.

#### Module 1.2: granulocyte activity

From the 16 genes grouped in this module, 14 are genes known to be expressed in granulocytes: 11 in neutrophils (BPI, CEACAM6, CEACAM8, DEFA4, ELANE, HP, MMP8, MPO, OLFM4, OLR1 and TCN1), two in eosinophils (RNASE2 and RNASE3) and one gene expressed in both cell-types (CRISP3). In addition, two of the seven genes in the neighboring 1.3 module are also expressed in neutrophils (ANXA3 and CD177). Several works have been published describing the role that neutrophils have both in multiple sclerosis and neuromyelitis optica, an autoimmune and inflammatory disease with lesions in the optical nerve and spinal cord that resembles multiple sclerosis [5]–[9]. According to a study published by Naegele et al in 2012 [10], neutrophils need to be in a primed state before being fully activated in a second step and the number of primed neutrophils is significantly higher in MS patients. Our results support the observations made by Naegele and colleagues, as we found that genes related to neutrophil activity are overexpressed in remission, some involved in degranulation among them (BPI, ELANE and DEFA4).

#### Module 2.1: regulatory non-coding RNAs

Most of the genes whose expression was found to be altered and are members of the female module 2.1 are non-coding regulatory RNAs (**Figure S2**). They are mostly involved in ribosomal RNA biogenesis and post-transcriptional control of gene expression. Although the intronic snoRNAs, most of the U-RNAs and the cajal-body specific RNA 4 (SCARNA4) are transcribed by RNA polymerase II, many of the RNA species involved in the altered regulatory network are transcribed by RNA polymerase III (POL III) [11]: 5S rRNA, an Y-RNA, VTRNA1-1, the snoRNAs of the SNORD3@ cluster [12] and the spliceosomal U6 RNAs (**Figure S5**). Notably, it has been described that the Epstein-Barr virus infection promotes transcriptional activity through the induction of the expression of all subunits of TFIIIC and BDP1 (a TFIIIB subunit), which are necessary for POLIII-mediated transcription initiation [13] and also induces the expression of MIR21 [14]. Based on that evidence, we propose that the deregulation of the genes of this module is a consequence of a reactivation of EBV B-cell infection happening in relapses of female patients.

#### Male module: olfactory receptors and cancer-related genes

The analysis of the proportion of the genes obtained from treatment-independent comparisons has revealed that this is the module that most accurately reflects the pathophysiological mechanisms of the disease (in males, in this particular case). However, it presents a high heterogeneity in respect to the biological role of its genes and, thus, it is the most difficult module to interpret. Nevertheless, the functional relations of some of the genes of this module with some of the events and cells known to be involved in multiple sclerosis as described by literature are shown in **figure 8**.

In 2012, Plessy et al confirmed Tbp, Ebf1 and Mef2 as transcription factors binding olfactory receptor (OR) gene promoters in mice [15]. Among these TFs, early B cell factor 1 (Ebf1) was previously described as having an important role in olfactory neuron development and differentiation in mice and rats [16]–[18]. Interestingly, EBF1 is essential for lineage specification in early B cell development in humans. Moreover, EBF1 seems to play an important role in EBNA2 mediated regulation of transcription that is critical for the conversion of resting B cells to a lymphoblastoid cell line (LCL). EBNA2 is a protein expressed by the Epstein - Barr virus in the latency program III and acts as the main viral transactivator, being essential for EBV-mediated growth transformation. It has also been proposed that the EBF binding site of the EBV LMP1 promoter is critical for the EBNA2-mediated activation [19]. In addition, the EBV-mediated immortalized B-lymphoblastoid cell lines with low permissivity for lytic reactivation show overexpression of EBF1 [20]. Thus, it could be hypothesized that, as with the female module 2.1, the deregulation of the genes of this module is a consequence of an EBV reactivation happening in relapses, characterized mainly by an overexpression of EBNA2.

### Gender Differences

Gender seems to strongly affect several aspects of both the immune system and MS. On one hand, the kinetics, magnitude and skewing of immune responses can differ dramatically between the sexes [21]. In general, females mount higher innate and adaptive immune responses than males, which can result in faster clearance of pathogens but also contributes to increased susceptibility to inflammatory and autoimmune diseases. [22].

On the other hand, gender appears to play critical roles in the development, progression and treatment of MS. As for the rest of autoimmune diseases, females have a higher risk of suffering MS and the female:male ratio has been increasing for the last 50 years [23] and is currently at 2.6∶1 [24]. Several genetic elements have also been found to confer risk for MS in a gender-specific manner [25], [26]. The molecular processes underlying the pathogenic events driving disease-progression seem also to be different in females and males and a clear gender bias has been detected in gene expression studies in MS [27], [28], suggesting that gender is a very important variable that needs to be taken into account when this kind of data are discussed.

Our results also support the notion that the molecular processes involved in multiple sclerosis, at least in its relapsing-remitting form, show remarkable differences between females and males. Although 31.85% of the genes of the main component of the coexpression network have been classified as not sex-specific (common genes), a deeper analysis of the results has revealed that, in fact, the modules classified as female-specific seem to have very little relevance in males and vice versa.

### EBV Gene Expression

In order to check the hypothesis of an EBV infection reactivation of B cells in relapse as a cause of the deregulation of the genes of the module 2.1 and the male module, the expression of lytic ant latency genes of the virus has been measured. When comparing the fold-changes between lytic and latency expression, no differences have been observed between conditions (controls, patients in remission and patients in relapse) in any of the groups (all samples, females and males). However, the lytic/latency gene expression pattern supports the idea of a viral reactivation in relapses of female patients, but not in male patients, as the pattern is just the opposite. Besides, EBNA2 gene expression does not show the *dowregulated in remission/upregulated in relapse* profile that we expected from the interpretation of the male module. The apparent female specificity of the EBV reactivation as an event triggering relapses can be explained by the fact that anti-EBNA1 antibody titers and HLA-DRB1*15∶01 have been shown to interact as risk factors in MS [29]–[31] and that HLA-DRB1*15∶01 has been suggested to confer risk just in female subjects [32].

Nevertheless, nothing conclusive can be said from the qPCR data obtained for the viral genes. Although the directions of the differences in the averages suggest that EBV infection reactivation may occur in relapses in female patients, no significant differences have been found in the statistical analysis. We believe the problem resides in the high number of PCR cycles necessary to detect the expression of viral genes in our RNA samples. The more cycles are used, more technical variability is introduced and getting good quality and stable results becomes more difficult. To address this problem, it will be necessary to perform further studies using RNA isolated from B cells with the aim of incrementing the presence of viral gene transcripts in the sample and lower the PCR cycles necessary to detect them.

### Relapsing-remitting Molecular Dynamics in Females

#### Potential role of vitamin B12

**Figure 9:**
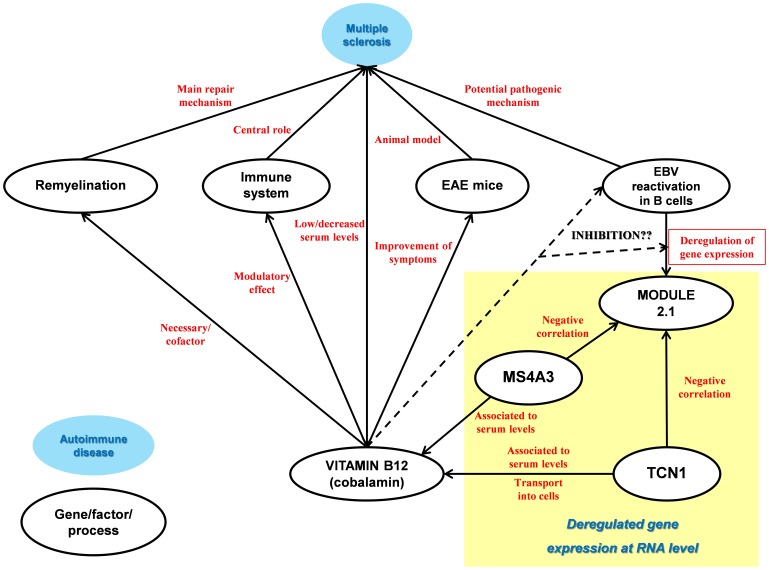
Functional relations between vitamin B12 and MS-related elements as described by literature along with the relevant gene expression regulation relations deduced from the coexpression network.

From the 2058 coexpression relations shown by the coexpression network, only 13 are negative correlations and 12 of them link 2 genes of the module 1.2, TCN1 and MS4A3, with several genes of the module 2.1. Coincidentally, these two genes are the only genes of the coexpression network that have been directly related to vitamin B12 physiology (**figure 9**) [33], [34]. Besides, the topological analysis of the female component has revealed that TCN1 is the most central gene of the network, suggesting it plays an important regulatory role.

Several pieces of evidence point towards a relation between vitamin B12, multiple sclerosis and remyalination [35], [36]. Moreover, the use of combination therapy with interferon-β and vitamin B12 has been shown to dramatically improve the clinical, histological and laboratory parameters in acute and chronic experimental autoimmune encephalomyelitis in SJL mice [37].

Besides, although the results of the ELISA show no differences in the Tcn1 serum concentration of healthy controls, patients in remission and patients in relapse, they confirm a significantly higher concentration in the samples from female patients with a CIS. These results support the idea of TCN1 being implicated in the pathophysiology of MS in female patients, at least at the initial stages of the disease. The discordance between the deregulation found at the RNA level and the lack of differences at the protein level could be explained by post-transcriptional effects exerted by treatments.

These results point towards a potential role of TCN1 and MS4A3 in the control of EBV reactivation and/or its effects on B lymphocytes through their role in vitamin B12 physiology. However, functional studies will have to be performed in order to confirm or reject this idea.

#### Control mechanisms vs. pathogenic mechanisms in females

The coexpression analysis has revealed two main coexpression modules related to pathophysiology in females. The module 1.2 is overexpressed in remission and underexpressed in relapse and it probably reflects the “primed” state that neutrophils have been shown to present in MS patients. The module 2.1, instead, is underexpressed in remission and overexpressed in relapse and evidence suggests that it may reflect the deregulation of host-cell gene expression produced by the Epstein-Barr virus infection in B lymphocytes. Besides, it has just been mentioned that two of the genes of the module 1.2, TCN1 and MS4A3, show negative correlation with several genes of the module 2.1, suggesting that they have some role in the control of EBV virus reactivation through their effect on vitamin B12 physiology.

**Figure 10:**
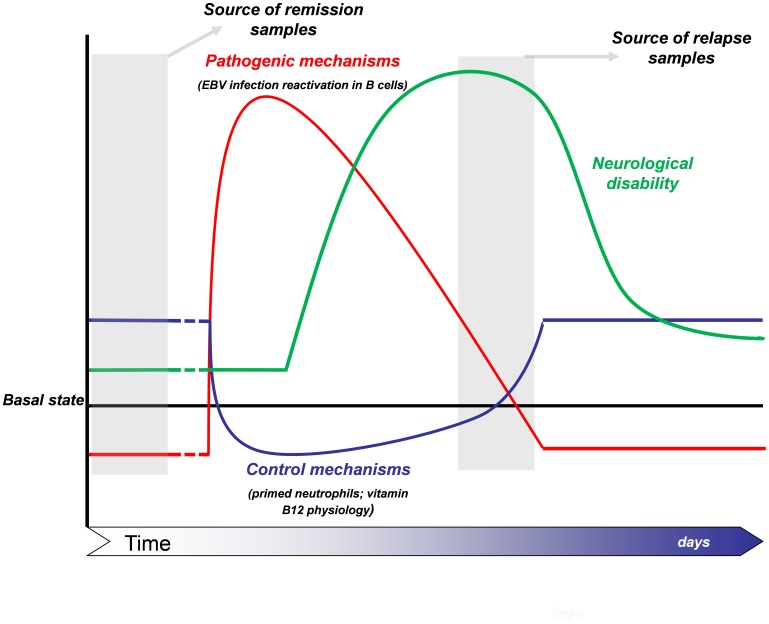
Theoretical model for the relapsing-remitting molecular dynamics responsible for disease progression of RRMS in female patients.

Based on this evidence, we propose a theoretical model for the relapsing-remitting molecular dynamics responsible for disease progression of RRMS in female patients (**figure 10**). According to this model, the primed state of neutrophils is part of the endogenous mechanisms aimed to keep under control the reactivation of EBV infection in the B cells; and its when control mechanisms fail that reactivation of EBV infection in B lymphocytes happens, triggering the chain of events leading to the pathogenic inflammatory response. In this model, we propose that the pathogenic processes triggered in peripheral blood act before the clinical symptoms appear and are nearly brought back to control when the neurological disability is at its highest, the precise moment where most of samples labeled as “relapse” were obtained.

### Limitations of the Study

We are aware that the present study has several limitations that, although do not diminish the validity of the results, must be taken into account when interpreting them. The necessity of subdividing the sample pool in order to obtain more homogeneous groups has forced us to work with small sample sizes in many comparisons. Thus, further studies with larger sample sizes are necessary in order to confirm or refute the validity of both our results and the hypotheses we pose regarding the total population of patients with MS. Besides, the cutoffs we have used in the statistical analyses are less stringent than the ones typical used in large-scale gene expression studies. However, the subsequent treatment of the data by matching the gene-lists obtained from different comparisons (if the probability of a gene appearing in list by chance is of p<0.01, the probability of this very gene appearing in two independent lists is p = 0.01×0.01 = 0.0001) and by creating the coexpression network (the probability of two genes with 68 expression values each presenting a Pearson’s correlation above |0.6| bay chance is 6.10^−8^) makes us very confident about the robustness that our final result, the coexpression network of differentially expressed genes, has from a statistical point of view.

Finally, our differential gene expression results have not been validated with another technique such as qPCR or Luminex technology. However, we consider that the systems biology approach used in this study makes the technical validation non-essential. The fact that the differentially expressed genes cluster in very tightly coexpressed modules makes very unlikely that the deregulation we detect for these genes is a consequence of stochasticity or technical artefacts. We consider that, in this particular case, the validation of the hypotheses we pose from the interpretation of the results (such as the relevance of TCN1 or the reactivation of EBV) is a more logical next step.

## Conclusions

The whole-genome gene expression analysis of peripheral blood leukocytes has allowed the observation that the genes that are differentially expressed in the two main phases of relapsing-remitting multiple sclerosis, relapse and remission, show a behavior that gives rise to a pattern we have called “mirror pattern”: if a gene is upregulated in remission is downregulated in relapse and vice versa. Besides, we have seen that the modules obtained in the coexpression analysis of these genes reflect molecular processes that are sex-specific. However, the literature-based interpretation of the results led us to the hypothesis of EBV infection reactivation having a central role in the pathogenic events in both male and female patients. Nevertheless, the EBV viral gene expression analysis results support that idea just in female patients and, thus, the reactivation of the virus seems not to happen in relapses of male patients with MS. Finally, the regulatory relations observed in the coexpression analysis suggest that the primed phenotype exhibited by neutrophils in female patients is part of the endogenous mechanisms aimed to keep under control the reactivation of EBV infection in B lymphocytes. The constant inflammatory environment that characterizes the peripheral blood of multiple sclerosis patients, thus, would represent, at least in female patients, a response of the body to control the pathogenic processes that act in relapse. More research is needed in order to deepen the understanding of the functional details and temporal dynamics of the molecular framework offered by the present study.

## Methods

### Subjects and Sample Collection

25 MS patients and 25 healthy controls were included in this study. Healthy control selection has been done trying to keep the patient and control groups as similar as possible regarding age and sex distribution (**table 3**; see **Table S4** for a complete description of each subject). All MS patients were diagnosed with Relapsing-Remitting Multiple Sclerosis according to McDonald criteria [38]. From each MS patient two blood samples were collected and analyzed, one sample in relapse and another one in remission; one sample was collected for each control subject, giving a total of 75 samples. Relapse was defined as the development of new or recurrent neurological symptoms not associated with fever or infection lasting for at least 24 hours and accompanied by new neurological signs, following a period of symptomatic stability of 30 days [39]. Peripheral blood of patients and healthy controls was obtained at the Neurology Department of Donostia University Hospital. Blood extraction was performed in the early morning for controls and remission samples and at arrival of the patient for relapse samples. In this last case, blood extraction always preceded the administration of steroids for relapses. RNA extraction was carried out no more than 2 hours after the blood was collected and during this time was kept at 4°C. In all the cases, 10 ml of blood were collected in EDTA tubes by venipuncture. All the procedures have been approved by the hospital’s ethic committee (*Comité ético de investigación Clínica del area sanitaria de Gipuzkoa*/Ethic committee of Clinical research in the Health area of Gipuzkoa ). Written informed consent was received from participants prior their inclusion in the study.

**Table 3:** Main characteristics of patients and controls included in the study.

	Patients	Controls
**Age (years)**		
*mean ± std*	39.94±10.73	37.88±8.66
*min − max*	23–66	23­57
**Sex**		
*Females*	14 (56%)	13 (52%)
*Males*	11 (44%)	12 (48%)
**EDSS**		
*mean ± std*	2.90±1.81	
*min − max*	0–7.5	
**Age at onset (years)**		
*mean ± std*	30.32±11.40	
*min − max*	11–65	
**Time of evolution (years)**		
*mean ± std*	9.62±7.96	
*min − max*	1–33	

### RNA Extraction

RNA extraction from leukocytes was performed using the LeucoLOCK™ Total RNA Isolation System by Ambion with the alternative protocol to recover Total RNA. RNA samples were aliquoted and stored at −80°C for later use. Samples were extracted and stored at the node of the Basque Biobank in Donostia - San Sebastián in accordance to their quality criteria (www.biobancovasco.com).

### Whole Genome Gene Expression

Whole genome gene expression of the samples was measured by the Human Gene 1.0 ST Affymetrix microarray. First, RNA integrity was checked with the Agilent RNA 6000 Nano kit and the samples with an RNA Integrity Value (RIN) above 6 were accepted to be further processed. 300 ng of total RNA were used for microarray analysis following the manufacturer’s instructions. Both the WT Expression Kit by Ambion and the GeneChip Hybridization Wash and Stain kit by Affymetrix were used in the process. Briefly, during the three-day protocol, complementary single-strand DNA was synthesized from RNA, to be later fragmented, labeled and hybridized during 16 hours. The hybridized microarrays were washed and stained in a GeneChip Fluidics Station 450 and scanned in a GeneChip 7G Scanner afterwards. Two of the relapse samples failed to provide any signal and, thus, the 73 files created from the scanning were stored for subsequent analysis.

### Data Analysis

#### Data normalization and quality control

The normalization of the data was performed using the Robust Multichip Algorithm (RMA) in the Expression Console software by Affymetrix. Then, several parameters including log-intensities of cell probes, Pearson’s correlation between arrays, positive vs negative control-probe intensity and perfect match vs background intensity were visualized in order to detect outlier samples that could introduce noise in the system. Finally, a Principal Component Analysis (PCA) was performed with the MEV 4.7.3 software [40], [41] to visualize the homo−/heterogeneity of the samples in a 2 dimensional-plot.

The PCA revealed a very high heterogeneity among the samples (**Figure S6A**) suggesting a high quantity of noise in the system. Some samples were removed from the analysis in order to reduce noise. However, a balance is needed to be kept between decreasing noise and reducing sample size and, thus, just the most obvious outliers and/or the least valuable samples were removed. Five samples were eliminated from further analysis: a control sample (lowest perfect match intensity/background intensity ratio), two remission samples with no paired relapse sample (a paired comparison could not be done) and a relapse/remission pair (biggest difference between mean intensities in the pair).

The “.cel” data from the remaining 68 samples were normalized with the RMA algorithm and the PCA analysis on these data showed a notable decrease in the heterogeneity of the samples (**Figure S6B**). The microarray data from this publication have been submitted to the NIH GEO repository (http://www.ncbi.nlm.nih.gov/geo/query/acc.cgi?acc=GSE41890) and assigned the identifier GSE41890 (username: haritzirizar; password: erduizti).

#### Differentially expressed gene lists

Multiple-testing correction makes the group-wise comparisons more stringent as more genes are included in the analysis. Therefore, we filtered the data to remove the least informative genes using the BRB-Array Tools software [BRB-ArrayTools Development Team, version 4.2.1] implemented in Microsoft Excel [Microsoft Corporation, Microsoft Office Professional Edition 2003]. First, the 50% of genes with lowest expression variability across samples were discarded. Second, the genes with a 90th percentile value smaller than 5.8 (the mean intensity of the negative control probes) were removed. After these two steps, 10828 out of the 33297 probesets remained for the subsequent analysis.

**Figure 11:**
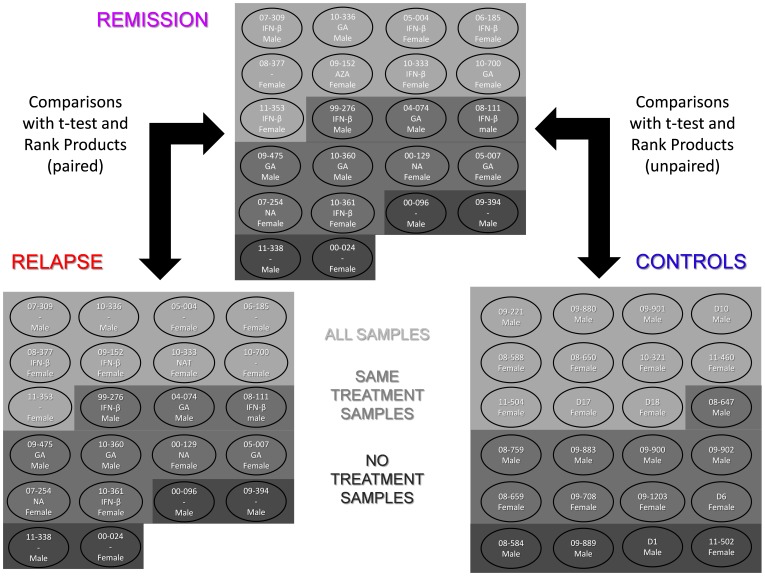
Distribution of samples across the subgroups made based on treatment, sex and condition. The code, sex and treatment-state are displayed for each subject (treatment is not displayed for controls). SAME TREATMENT SAMPLES: samples of patients with the same treatment state in relapse and remission and matched controls; NO TREATMENT SAMPLES: samples of patients with no treatment in relapse and remission and matched controls; AZA: azathioprine; GA: glatiramer acetate; IFN-β: interferon β; NA: natalizumab.

Differentially expressed gene lists were obtained using the MEV 4.7.3 software [40], [41]. Several data subdivisions based on sex and treatment were done (**figure 11**). The reason for the sex-based subdivision is that this variable has a strong influence both in the risk of suffering the disease and its progression [25], [42]. On the other hand, multiple sclerosis modifying treatments have been shown to affect strongly peripheral blood gene expression [43] and, thus, we decided to perform some comparisons with a smaller influence of treatment. Two comparisons, relapse vs remission and remission vs controls, were made for each subgroup. All the comparisons were performed twice, one using a t-test and another using the Rank Products [44], for a total of 36 comparisons.

As the system proved to be very noisy, the differential expression analysis was performed in a non-stringent manner and p<0.01 (uncorrected) and FDR <0.1 were set as thresholds for the t-tests and the Rank Products, respectively. Anyway, in order to detect the most relevant genes, lists with the genes that appeared to be differentially expressed in both comparisons (relapse vs remission/remission vs controls) were created (**Figure S1**), based on the assumption that if a gene is altered in both conditions (relapse/remission) it should have some pathological relevance. This procedure resulted in 18 lists with genes deregulated in both conditions.

#### Coexpression analysis

The coexpression analysis of the data was performed using both the Python programming language and the Cytoscape [45] network visualization program. A correlation matrix was created for all the samples based on the expression of the differentially expressed genes. A vector was defined for each gene with the gene’s expression values for X subjects (68) and a pair-wise coexpression matrix of size NxN was constructed by computing the correlation between the two vectors associated with each gene-pair, N being the number of differentially expressed genes. As the thresholds used at the differential expression analysis were very loose, a genome-wide significance threshold (p<6. 10^−8^) was used to establish the cutoff Pearson’s R value (R>|0.6|) to cut the correlation matrix. The resulting network was visualized in Cytoscape.

### Tcn1 Concentration in Serum

The concentration of the protein product of transcobalamin 1 (Tcn1) has been measured in the serum of a initial set of 49 samples (17 healthy controls, 18 patients in remission, 10 patients in relapse and 4 subjects with Clinically Isolated Syndrome (CIS)) and a second set of 19 serum samples (12 healthy subjects and 7 new patients with CIS). In the initial sample set, 26 out of the 49 sera were obtained from the very same peripheral blood samples from which RNA was isolated for the microarray analysis. Besides, from the 11 patients in CIS included in the analysis, 8 had already progressed to definite MS at the moment of the study. A commercial ELISA kit was used (Uscn Life Science Inc., Wuhan, Hubei, China) for Tcn1 concentration measurements in serum samples and the experiment was done following the manufacturer’s instructions. The best dilution of the sera was found to be 1∶1. Measurements were done in duplicate for the initial sample set and in triplicate for the second sample set. The R values of the lineal regressions of the standard curve lied between 0.976 and 0.997. A Kolmogorov-Smirnov test was performed on the two datasets and, as none of the two distributions was found to be significantly different from a normal distribution (p>0.05 in both tests), a Student’s t-test was used to obtain the p values of the comparison of the averages.

### EBV Gene Expression

The expression of 5 viral genes, BZLF1, EBNA1, EBNA2, LMP1 and LMP2, has been measured by qPCR in 50 out of the 68 samples from the study (15 patients in remission, 16 patients in relapse and 19 healthy controls) and in three RNA samples obtained from human fibroblasts that were used as negative controls. cDNA was obtained by RT-PCR with the High Capacity cDNA Reverse Transcription kit (Life Technologies, Carlsbad, CA, US) and 25 µl of RNA (at 25 ng/µl, 1250 ng in total) were used as input. As the expression of these genes is very low, a pre-amplification step was necessary for their detection. The pre-amplification PCR was done for each of the 5 genes, using 3 µl of cDNA. For the qPCR, 2.5 µl of the pre-amp PCR product were used. The quantitative PCR was performed on a 7900HT Fast Real-Time PCR System (Life Technologies, Carlsbad, CA, US). [46]. A negative expression threshold for each gene was obtained by averaging the Ct values obtained from fibroblasts and subtracted from the –Ct values of the samples for each gene to obtain a –dCt (all the –dCts below 0 were considered not to have significant expression and were set at 0).

## Supporting Information

Figure S1 **Match-ups of the differentially expressed gene-lists obtained from each of the relapse vs. remission and remission vs. controls comparison pairs, for t-test (A) and Rank Products (B) comparisons.**
(TIF)

Figure S2 **Biological information of the 4 biggest modules of the coexpression network.** In the fold-change box-plots, the terms “Remission” and “Relapse” correspond to the “remission vs. controls” and “relapse vs. remission” comparisons, respectively. The p value of the biological term enrichment analysis is a non-corrected value.
(TIF)

Figure S3 **Scatter plot of the significance and deviation of the genes of each module in the different data subsets.** Data subsets have been created based on treatment state (different treatment samples, same treatment samples and no treatment samples) and sex (females/males/all) and two parameters have been calculated for each gene: **1– mean p^2^** and the **mean accumulated deviation**. **p^2^** = (p value _relapse vs. remission_) **x** (p value _remission vs. controls_); **Accumulated deviation** = |1– FC _relapse vs. remission_|+|1– FC _remission vs. controls_|. The name of the data points corresponds to **Module_Treatment state_Sex**.
(TIF)

Figure S4 **Importance of the genes in the topology of the female component as measured by their betweeness-centrality, which is visualized by node size.**
(TIF)

Figure S5 **Regulatory network of the RNA species found to be altered in the female module 2.1 and the relations of some of the elements of the network with several autoimmune diseases as described in literature.**
(TIF)

Figure S6 Results of the Principal Component Analysis based on the expression of the 33297 probesets of the Human Gene array showing the distribution of the samples on the 2 principal components before (A, 73 samples) and after (B, 68 samples) the exclusion of the 5 samples. As can be observed in the values of the two axes a great reduction in heterogeneity and system noise was achieved by the removal of the samples.
(TIF)

Table S1 **List of common and male genes and their fold-changes for each comparison.** (Gene symbol/given name: male genes in red, common genes in purple; **REL.**: relapse vs remission; **REM.**: remission vs controls; **cDNA**: unkown cDNA; **mtRNA**: mitocondrial RNA; **ncRNA**: non-codingRNA; **rRNA**: ribosomal RNA; **^1^**CCL4/CCL4L1/CCL4L2; **^2^**CNTNAP3/CNTNAP3B; **^3^**RABGGTB/SNORD45B; **^4^**TAF1D/SNORA40; **^5^**GAGE8/GAGE7/GAGE12I/GAGE12G/GAGE12F/GAGE12B/GAGE6/GAGE12J/GAGE2E/GAGE13/GAGE12H; **^6^**OR11H12/OR11H1/OR11H2; **^7^**OR2M1P/OR2M7/OR2M3/OR2M2/OR2M5; **^8^**OR4F17/OR4F4/OR4F5; **^9^**OR4F3/OR4F16/OR4F29/OR4F21; **^10^**ROCK1P1/ROCK1).
(XLS)

Table S2 **List of female genes and their fold-changes for each comparison. (REL**.: relapse vs remission; **REM.**: remission vs controls; **cDNA**: unkown cDNA; **mtRNA**: mitocondrial RNA; **ncRNA**: non-coding RNA; **rRNA**: ribosomal RNA; **^1^**C7orf40/SNORA9; **^2^**RPSA/SNORA62; **^3^**USP18/USP41).
(XLS)

Table S3 **List of genes of the 4 biggest modules of the coexpression network with the fold-changes of remission and relapse and the specificity assigned to each gene.**
(DOCX)

Table S4 **Main characteristics of the subjects involved in the study. EDSS: Expanded Disability Status Scale.**
(DOCX)
