# Supplementary figures and images for: Transcriptomic Profile Reveals Gender-Specific Molecular Mechanisms Driving Multiple Sclerosis Progression
Source: PLoS One. 2014 Feb 28;9(2):e90482. doi: 10.1371/journal.pone.0090482 (PMC3938749; doi:10.1371/journal.pone.0090482)

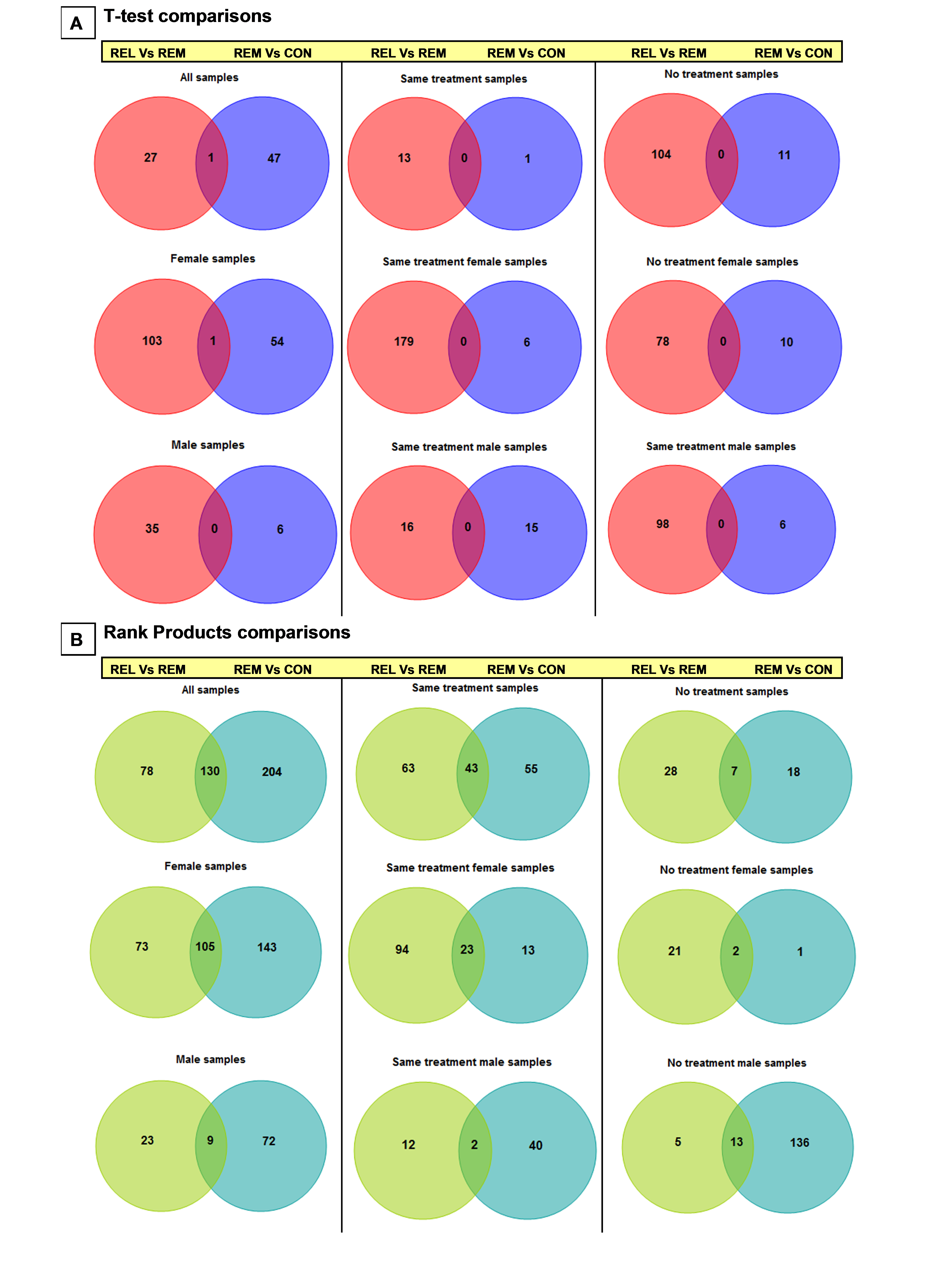

Supplement: Figure S1 — Match-ups of the differentially expressed gene-lists obtained from each of the relapse vs. remission and remission vs. controls comparison pairs, for t-test (A) and Rank Products (B) comparisons. (TIF) [file pone.0090482.s001.tif]

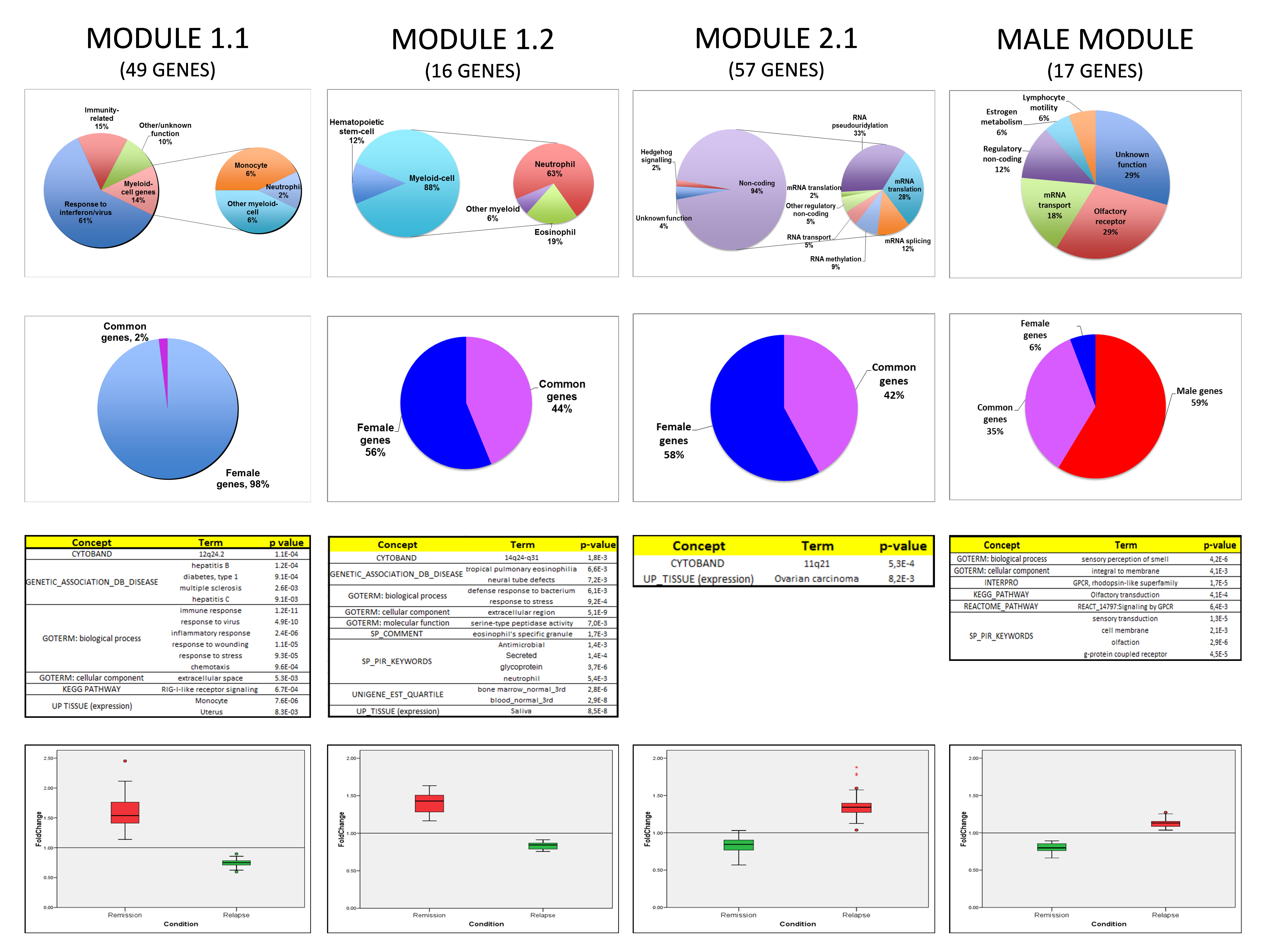

Supplement: Figure S2 — Biological information of the 4 biggest modules of the coexpression network. In the fold-change box-plots, the terms “Remission” and “Relapse” correspond to the “remission vs. controls” and “relapse vs. remission” comparisons, respectively. The p value of the biological term enrichment analysis is a non-corrected value. (TIF) [file pone.0090482.s002.tif]

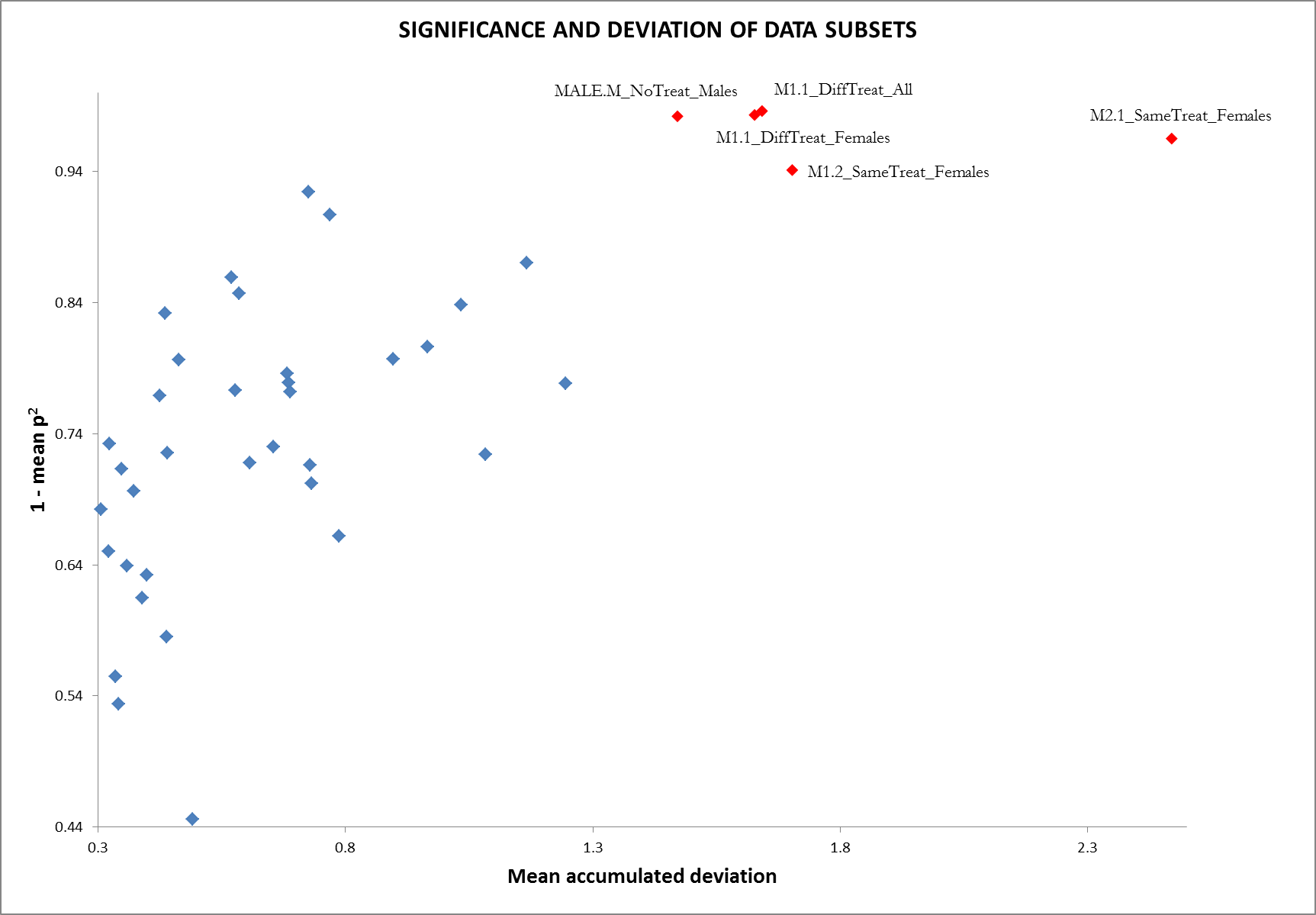

Supplement: Figure S3 — Scatter plot of the significance and deviation of the genes of each module in the different data subsets. Data subsets have been created based on treatment state (different treatment samples, same treatment samples and no treatment samples) and sex (females/males/all) and two parameters have been calculated for each gene: 1– mean p2 and the mean accumulated deviation. p2 = (p value relapse vs. remission) x (p value remission vs. controls); Accumulated deviation = |1– FC relapse vs. remission|+|1– FC remission vs. controls|. The name of the data points corresponds to Module_Treatment state_Sex. (TIF) [file pone.0090482.s003.tif]

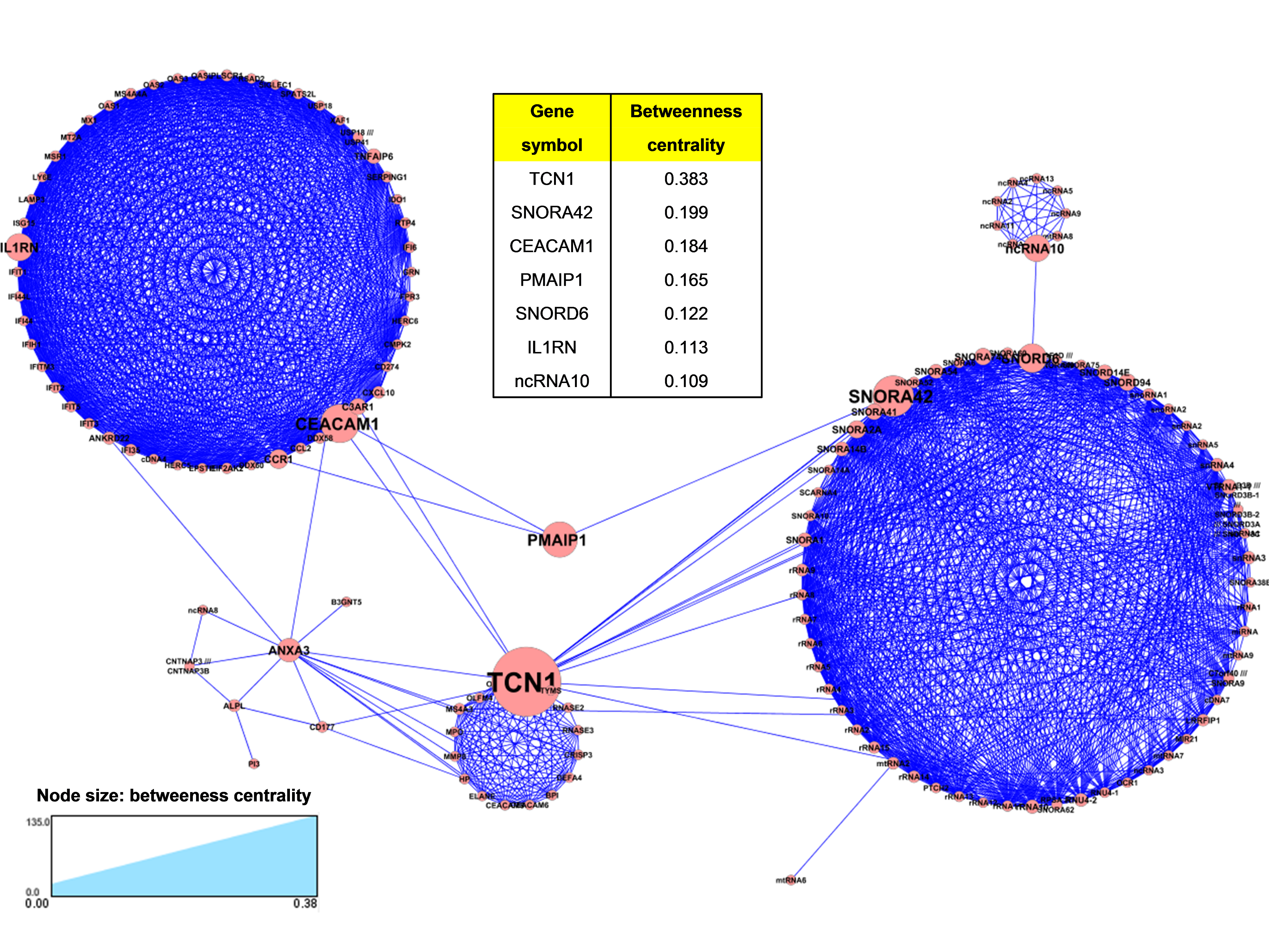

Supplement: Figure S4 — Importance of the genes in the topology of the female component as measured by their betweeness-centrality, which is visualized by node size. (TIF) [file pone.0090482.s004.tif]

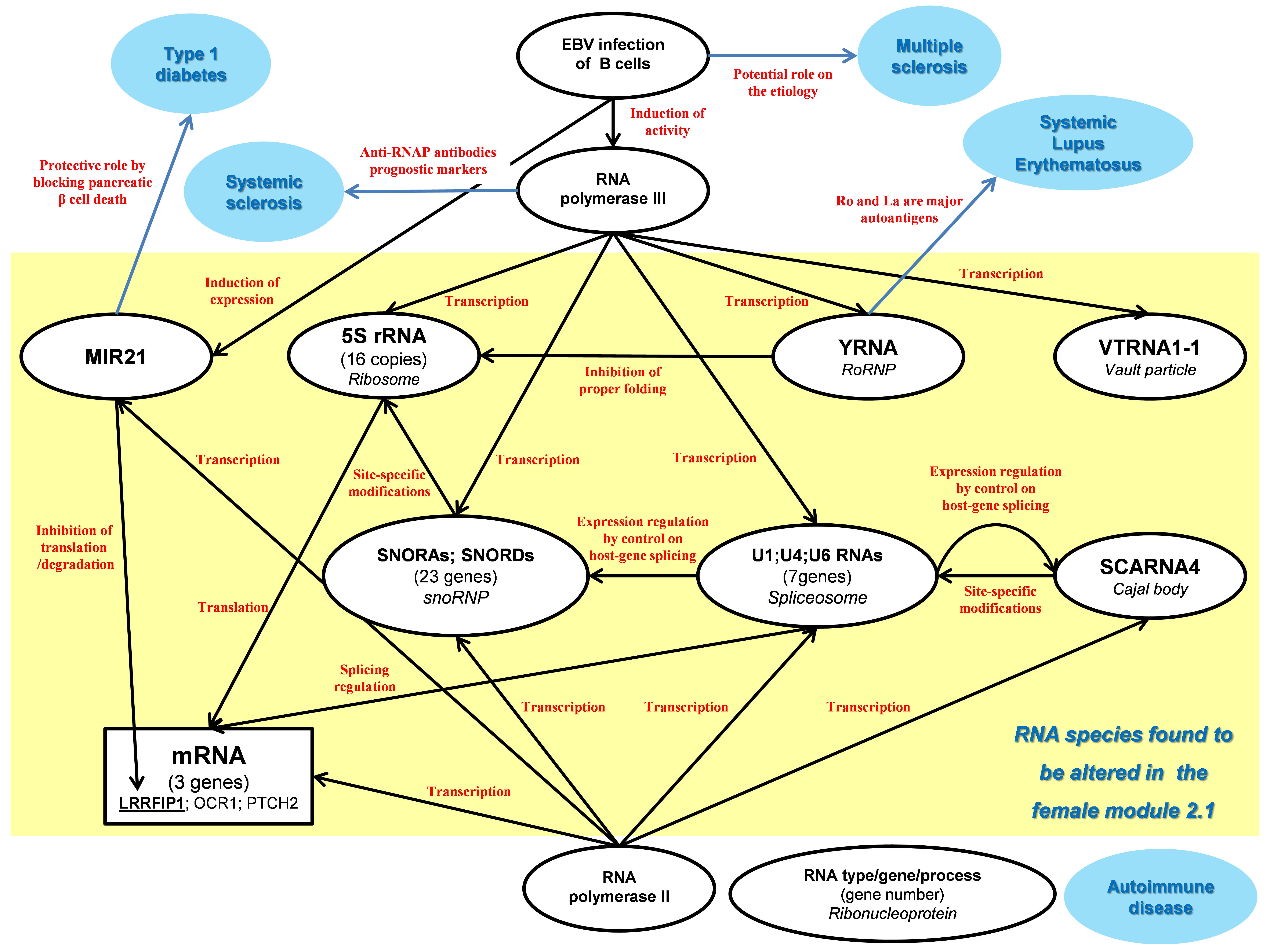

Supplement: Figure S5 — Regulatory network of the RNA species found to be altered in the female module 2.1 and the relations of some of the elements of the network with several autoimmune diseases as described in literature. (TIF) [file pone.0090482.s005.tif]
