# Supplementary material for: Transcriptomic Profile Reveals Gender-Specific Molecular Mechanisms Driving Multiple Sclerosis Progression
Source: PLoS One. 2014 Feb 28;9(2):e90482. doi: 10.1371/journal.pone.0090482 (PMC3938749; doi:10.1371/journal.pone.0090482)
Supplement: Table S3 — List of genes of the 4 biggest modules of the coexpression network with the fold-changes of remission and relapse and the specificity assigned to each gene. (DOCX) [file pone.0090482.s009.docx]

| **FEMALE COMPONENT** | | | | | | | | | | | | | | |
| --- | --- | --- | --- | --- | --- | --- | --- | --- | --- | --- | --- | --- | --- | --- |
| **MODULE 1.1** | | | | | **MODULE 1.2** | | | | | **MODULE 2.1** | | | | |
| **Affymetrix** | **Gene symbol/** | **Fold-change** | | **Specificity** | **Affymetrix** | **Gene symbol/** | **Fold-change** | | **Specificity** | **Affymetrix** | **Gene symbol/** | **Fold-change** | | **Specificity** |
| **Probeset ID** | **given name** | **REMvs.CON** | **RELvs.REM** |  | **Probeset ID** | **given name** | **REMvs.CON** | **RELvs.REM** |  | **Probeset ID** | **given name** | **REMvs.CON** | **RELvs.REM** |  |
| 8007620 | GRN | 1.139 | 0.897 | Female | 7973110 | RNASE2 | 1.165 | 0.784 | Common | 8073680 | tRNA | 0.569 | 1.877 | Common |
| 8146092 | IDO1 | 1.200 | 0.717 | Common | 7940216 | MS4A3 | 1.181 | 0.909 | Female | 8108627 | VTRNA1-1 | 0.649 | 1.773 | Female |
| 8030866 | FPR3 | 1.227 | 0.844 | Female | 8019842 | TYMS | 1.273 | 0.843 | Common | 7920875 | SCARNA4 | 0.704 | 1.597 | Female |
| 7960874 | C3AR1 | 1.296 | 0.787 | Female | 7973105 | RNASE3 | 1.277 | 0.757 | Female | 7910377 | RN5S_1 | 0.715 | 1.324 | Common |
| 8149448 | MSR1 | 1.319 | 0.732 | Female | 8024056 | ELANE | 1.290 | 0.864 | Common | 7920873 | SNORA42 | 0.718 | 1.324 | Female |
| 8086600 | CCR1 | 1.344 | 0.856 | Female | 8016932 | MPO | 1.385 | 0.913 | Female | 8005547 | SNORD3* | 0.724 | 1.247 | Female |
| 7940237 | MS4A4A | 1.350 | 0.757 | Female | 8062444 | BPI | 1.402 | 0.843 | Female | 8107857 | RN5S_13 | 0.724 | 1.323 | Common |
| 8092348 | LAMP3 | 1.379 | 0.729 | Female | 8126905 | CRISP3 | 1.424 | 0.796 | Common | 7967028 | RNU4-2 | 0.731 | 1.877 | Common |
| 8095376 | MT2A | 1.390 | 0.764 | Female | 7969288 | OLFM4 | 1.435 | 0.913 | Female | 8014704 | RN5S_7 | 0.738 | 1.431 | Female |
| 8154233 | CD274 | 1.392 | 0.765 | Female | 7948444 | TCN1 | 1.458 | 0.784 | Female | 8133688 | SNORA14A | 0.738 | 1.152 | Female |
| 8160559 | DDX58 | 1.398 | 0.832 | Female | 8037222 | CEACAM8 | 1.459 | 0.862 | Female | 7996260 | RN5S_6 | 0.743 | 1.547 | Female |
| 8037205 | CEACAM1 | 1.402 | 0.849 | Female | 7961142 | OLR1 | 1.503 | 0.790 | Common | 7937483 | SNORA52 | 0.749 | 1.381 | Female |
| 8162531 | MT1P1 | 1.411 | 0.721 | Female | 8029098 | CEACAM6 | 1.510 | 0.873 | Common | 8168079 | RN5S_16 | 0.750 | 1.328 | Female |
| 8045688 | TNFAIP6 | 1.430 | 0.702 | Female | 7997188 | HP | 1.512 | 0.838 | Common | 8031931 | RN5S_9 | 0.750 | 1.506 | Common |
| 8084732 | RTP4 | 1.441 | 0.751 | Female | 7951246 | MMP8 | 1.524 | 0.787 | Female | 8062490 | SNORA60 | 0.768 | 1.343 | Female |
| 8007446 | IFI35 | 1.448 | 0.782 | Female | 8149109 | DEFA4 | 1.633 | 0.848 | Female | 7938625 | RN5S_4 | 0.769 | 1.394 | Female |
| 7934898 | ANKRD22 | 1.454 | 0.808 | Female |  |  |  |  |  | 7962827 | SNORA2A | 0.774 | 1.424 | Female |
| 7958913 | OAS2 | 1.462 | 0.785 | Female |  |  |  |  |  | 8050350 | RN5S_10 | 0.778 | 1.375 | Female |
| 7896817 | ISG15 | 1.471 | 0.770 | Female |  |  |  |  |  | 8016213 | RN5S_8 | 0.781 | 1.278 | Common |
| 8103563 | DDX60 | 1.473 | 0.809 | Female |  |  |  |  |  | 7925182 | SNORA14B | 0.789 | 1.384 | Female |
| 8096335 | HERC6 | 1.474 | 0.770 | Female |  |  |  |  |  | 8041204 | SNORA10 | 0.805 | 1.334 | Female |
| 7929072 | IFIT5 | 1.479 | 0.752 | Female |  |  |  |  |  | 7919560 | RNU1* | 0.808 | 1.341 | Female |
| 8044574 | IL1RN | 1.504 | 0.722 | Female |  |  |  |  |  | 8130622 | RN5S_14 | 0.814 | 1.298 | Female |
| 8101126 | CXCL10 | 1.508 | 0.626 | Female |  |  |  |  |  | 8108420 | SNORA74A | 0.826 | 1.319 | Female |
| 8056285 | IFIH1 | 1.509 | 0.761 | Female |  |  |  |  |  | 8009380 | SNORA38B | 0.831 | 1.191 | Female |
| 7940028 | SERPING1 | 1.567 | 0.720 | Female |  |  |  |  |  | 8059708 | SNORA75 | 0.837 | 1.441 | Female |
| 7958884 | OAS1 | 1.574 | 0.756 | Female |  |  |  |  |  | 7961418 | RN5S_5 | 0.839 | 1.271 | Common |
| 8064716 | SIGLEC1 | 1.588 | 0.775 | Female |  |  |  |  |  | 8043276 | SNORD94 | 0.844 | 1.382 | Female |
| 8091327 | PLSCR1 | 1.608 | 0.719 | Female |  |  |  |  |  | 8078918 | SNORA62 | 0.845 | 1.313 | Female |
| 8004184 | XAF1 | 1.609 | 0.770 | Female |  |  |  |  |  | 8087780 | RN5S_11 | 0.851 | 1.163 | Common |
| 8051501 | EIF2AK2 | 1.620 | 0.747 | Female |  |  |  |  |  | 7917468 | RN5S_2 | 0.852 | 1.142 | Common |
| 7929047 | IFIT2 | 1.664 | 0.821 | Female |  |  |  |  |  | 7932792 | RN5S_3 | 0.863 | 1.125 | Common |
| 7914127 | IFI6 | 1.678 | 0.710 | Female |  |  |  |  |  | 7915612 | PTCH2 | 0.864 | 1.355 | Common |
| 8047272 | SPATS2L | 1.694 | 0.657 | Female |  |  |  |  |  | 7951032 | SNORA1 | 0.883 | 1.491 | Female |
| 8050102 | CMPK2 | 1.755 | 0.707 | Female |  |  |  |  |  | 7967030 | RNU4-1 | 0.888 | 1.792 | Female |
| 7945371 | IFITM3 | 1.757 | 0.797 | Female | **MALE COMPONENT** | | | | | 8116952 | RNU1-11P | 0.888 | 1.387 | Female |
| 8006433 | CCL2 | 1.763 | 0.596 | Female |  |  |  |  |  | 7951038 | SNORA40 | 0.893 | 1.171 | Common |
| 8148572 | LY6E | 1.775 | 0.650 | Female | **MALE MODULE** | | | | | 7932635 | RNU6*_2 | 0.896 | 1.319 | Common |
| 7967117 | OASL | 1.806 | 0.735 | Female | **Affymetrix** | **Gene symbol/** | **Fold-change** | | **Specificity** | 8047780 | SNORA41 | 0.896 | 1.343 | Common |
| 7929052 | IFIT3 | 1.813 | 0.782 | Female | **Probeset ID** | **given name** | **REMvs.CON** | **RELvs.REM** |  | 7952335 | SNORD14E | 0.899 | 1.199 | Female |
| 8068713 | MX1 | 1.834 | 0.740 | Female | 8100310 | cDNA_5 | 0.662 | 1.144 | Male | 8135943 | RN5S_15 | 0.900 | 1.404 | Common |
| 8096361 | HERC5 | 1.909 | 0.679 | Female | 8100758 | UGT2B7 | 0.694 | 1.085 | Common | 7945801 | SNORA54 | 0.902 | 1.132 | Female |
| 7958895 | OAS3 | 1.926 | 0.714 | Female | 8148962 | OR4F*2 | 0.714 | 1.254 | Male | 8096459 | RN5S_12 | 0.903 | 1.037 | Common |
| 7971296 | EPSTI1 | 1.926 | 0.749 | Female | 8161440 | YRNA_11 | 0.732 | 1.192 | Common | 8045533 | mit_tRNA_3 | 0.926 | 1.205 | Common |
| 7929065 | IFIT1 | 1.986 | 0.683 | Female | 8023937 | OR4F*1 | 0.761 | 1.111 | Male | 8117018 | RNU6*_1 | 0.930 | 1.453 | Female |
| 8074606 | USP18 | 2.016 | 0.655 | Female | 8074194 | OR11H* | 0.765 | 1.088 | Male | 8154207 | mit_tRNA_6 | 0.931 | 1.207 | Common |
| 7902553 | IFI44 | 2.039 | 0.636 | Female | 7970392 | cDNA_2 | 0.773 | 1.273 | Common | 8139456 | SNORA9 | 0.937 | 1.574 | Female |
| 7902541 | IFI44L | 2.113 | 0.660 | Female | 8043502 | mit_tRNA_2 | 0.782 | 1.137 | Common | 7908861 | OCR1 | 0.943 | 1.357 | Common |
| 8040080 | RSAD2 | 2.452 | 0.624 | Female | 8095005 | cDNA_3 | 0.798 | 1.077 | Male | 8049530 | LRRFIP1 | 0.949 | 1.349 | Common |
|  |  |  |  |  | 8053427 | YRNA_5 | 0.813 | 1.164 | Male | 8078916 | SNORA6 | 0.956 | 1.147 | Female |
|  |  |  |  |  | 8100308 | cDNA_4 | 0.816 | 1.036 | Common | 7928489 | YRNA_3 | 0.968 | 1.371 | Common |
|  |  |  |  |  | 8117718 | OR2J3 | 0.831 | 1.101 | Common | 8008885 | MIR21 | 0.972 | 1.352 | Common |
|  |  |  |  |  | 8167560 | GAGE* | 0.854 | 1.153 | Male | 7951030 | SNORD6 | 0.976 | 1.273 | Female |
|  |  |  |  |  | 7992893 | mit_tRNA_1 | 0.869 | 1.131 | Female | 8150862 | SNORA1 | 0.984 | 1.346 | Female |
|  |  |  |  |  | 7911260 | OR2M* | 0.881 | 1.077 | Male | 8130181 | RNU4-7P | 0.986 | 1.396 | Female |
|  |  |  |  |  | 8019804 | ROCK1 | 0.886 | 1.141 | Male | 7958207 | cDNA_1 | 1.007 | 1.277 | Common |
|  |  |  |  |  | 8083469 | mit_tRNA_4 | 0.892 | 1.052 | Male | 7941563 | SNORD13 | 1.031 | 1.288 | Common |
