# Supplementary material for: Transcriptomic Profile Reveals Gender-Specific Molecular Mechanisms Driving Multiple Sclerosis Progression
Source: PLoS One. 2014 Feb 28;9(2):e90482. doi: 10.1371/journal.pone.0090482 (PMC3938749; doi:10.1371/journal.pone.0090482)
Supplement: Table S4 — Main characteristics of the subjects involved in the study. EDSS: Expanded Disability Status Scale. (DOCX) [file pone.0090482.s010.docx]

| **Sample ID** | **Condition** | **Sex** | **Age (years)** | **EDSS** | **MS treatment** | **Age at onset (years)** | **Evolution time (years)** |
| --- | --- | --- | --- | --- | --- | --- | --- |
| 00-024 | Relapse | Female | 47 | 7.5 | - | 27 | 22 |
|  | Remission |  | 48 | 7.5 | - |  | 23 |
| 00-129 | Relapse | Female | 28 | 3.5 | Tysabri | 15 | 12 |
|  | Remission |  | 29 | 3.5 | Tysabri |  | 13 |
| 05-004 | Relapse | Female | 32 | 1 | - | 11 | 19 |
|  | Remission |  | 30 | 0 | Betaferon |  | 17 |
| 05-007 | Relapse | Female | 35 | 0 | Copaxone | 21 | 13 |
|  | Remission |  | 34 | 0 | Copaxone |  | 12 |
| 06-185 | Relapse | Female | 36 | 2 | - | 30 | 6 |
|  | Remission |  | 36 | 3-3.5 | Rebif44 |  | 3 |
| 07-254 | Relapse | Female | 45 | 3 | Tysabri | 40 | 4 |
|  | Remission |  | 44 | 3.5 | Tysabri |  | 3 |
| 08-377 | Relapse | Female | 50 | 3.5 | Avonex | 43 | 7 |
|  | Remission |  | 50 | 3.5 | - |  | 7 |
| 09-1166 | Relapse | Female | 65 | 2.5 | - | 65 | 0 |
|  | Remission |  | 66 | 2 | Rebif 44 |  | 1 |
| 09-152 | Relapse | Female | 47 | 4.5 | Betaferon | 43 | 13 |
|  | Remission |  | 49 | 4 | Imurel |  | 14 |
| 09-238 | Relapse | Female | 34 | 0 | - | 33 | 0 |
|  | Remission |  | 36 | 0 | Copaxone |  | 2 |
| 10-333 | Relapse | Female | 46 | 4.5 | Tysabri | 35 | 20 |
|  | Remission |  | 45 | 5 | Rebif44 |  | 19 |
| 10-361 | Relapse | Female | 38 | 3 | Betaferon | 18 | 9 |
|  | Remission |  | 39 | 3 | Betaferon |  | 10 |
| 10-700 | Relapse | Female | 29 | 2 | - | 22 | 6 |
|  | Remission |  | 30 | 2 | Copaxone |  | 7 |
| 11-353 | Relapse | Female | 29 | 3.5 | - | 24 | 5 |
|  | Remission |  | 29 | 3 | Rebif44 |  | 5 |
| 00-096 | Relapse | Male | 58 | 6 | - | 25 | 33 |
|  | Remission |  | 59 | 6 | - |  | 33 |
| 04-074 | Relapse | Male | 43 | 2 | Copaxone | 29 | 14 |
|  | Remission |  | 43 | 2 | Copaxone |  | 14 |
| 06-186 | Relapse | Male | 28 | 4 | Copaxone | 24 | 3 |
|  | Remission |  | 31 | 4 | Tysabri |  | 6 |
| 07-309 | Relapse | Male | 29 | 3 | - | 26 | 2 |
|  | Remission |  | 29 | 3 | Rebif 44 |  | 2 |
| 08-111 | Relapse | Male | 48 | 0 | Avonex | 24 | 14 |
|  | Remission |  | 48 | 0 | Avonex |  | 14 |
| 09-394 | Relapse | Male | 43 | 2 | - | 43 | 0 |
|  | Remission |  | 44 | 1 | - |  | 1 |
| 09-475 | Relapse | Male | 40 | 4 | Copaxone | 32 | 8 |
|  | Remission |  | 40 | 4 | Copaxone |  | 7 |
| 10-336 | Relapse | Male | 23 | 3.5 | - | 22 | 0 |
|  | Remission |  | 23 | 2.5 | Copaxone |  | 1 |
| 10-360 | Relapse | Male | 34 | 2 | Copaxone | 24 | 8 |
|  | Remission |  | 35 | 0 | Copaxone |  | 9 |
| 11-338 | Relapse | Male | 41 | 2.5 | - | 39 | 1 |
|  | Remission |  | 41 | 2.5 | - |  | 1 |
| 99-276 | Relapse | Male | 39 | 4 | Betaferon | 36 | 12 |
|  | Remission |  | 38 | 3.5 | Betaferon |  | 12 |
| 08-588 | Control | Female | 34 |  |  |  |  |
| 08-650 | Control | Female | 23 |  |  |  |  |
| 08-659 | Control | Female | 35 |  |  |  |  |
| 09-1203 | Control | Female | 29 |  |  |  |  |
| 09-171 | Control | Female | 49 |  |  |  |  |
| 09-708 | Control | Female | 40 |  |  |  |  |
| 10-321 | Control | Female | 35 |  |  |  |  |
| 11-460 | Control | Female | 33 |  |  |  |  |
| 11-502 | Control | Female | 48 |  |  |  |  |
| 11-504 | Control | Female | 30 |  |  |  |  |
| D17 | Control | Female | 31 |  |  |  |  |
| D18 | Control | Female | 36 |  |  |  |  |
| D6 | Control | Female | 44 |  |  |  |  |
| 08-584 | Control | Male | 57 |  |  |  |  |
| 08-647 | Control | Male | 33 |  |  |  |  |
| 08-759 | Control | Male | 42 |  |  |  |  |
| 09-221 | Control | Male | 29 |  |  |  |  |
| 09-880 | Control | Male | 24 |  |  |  |  |
| 09-883 | Control | Male | 37 |  |  |  |  |
| 09-899 | Control | Male | 43 |  |  |  |  |
| 09-900 | Control | Male | 48 |  |  |  |  |
| 09-901 | Control | Male | 50 |  |  |  |  |
| 09-902 | Control | Male | 45 |  |  |  |  |
| D1 | Control | Male | 41 |  |  |  |  |
| D10 | Control | Male | 31 |  |  |  |  |
